# Xylan Is Critical for Proper Bundling and Alignment of Cellulose Microfibrils in Plant Secondary Cell Walls

**DOI:** 10.3389/fpls.2021.737690

**Published:** 2021-09-23

**Authors:** Jacob D. Crowe, Pengchao Hao, Sivakumar Pattathil, Henry Pan, Shi-You Ding, David B. Hodge, Jacob Krüger Jensen

**Affiliations:** ^1^Department of Chemical Engineering & Materials Science, Michigan State University, East Lansing, MI, United States; ^2^Department of Chemistry, Michigan State University, East Lansing, MI, United States; ^3^Complex Carbohydrate Research Center, The University of Georgia, Athens, GA, United States; ^4^Department of Chemical Engineering, University of Texas, Austin, TX, United States; ^5^Department of Plant Biology, Michigan State University, East Lansing, MI, United States; ^6^Department of Energy Great Lakes Bioenergy Research Center, Michigan State University, East Lansing, MI, United States; ^7^Department of Chemical & Biological Engineering, Montana State University, Bozeman, MT, United States; ^8^Section for Plant Glycobiology, Department of Plant and Environmental Sciences, University of Copenhagen, Copenhagen, Denmark

**Keywords:** xylan (hemicellulose), glucuronoxylan, atomic force micorscopy (AFM), secondary cell wall (SCW), cell wall mechanical properties, irregular xylan mutants (*irx*), cellulose deposition, cellulose arrangement

## Abstract

Plant biomass represents an abundant and increasingly important natural resource and it mainly consists of a number of cell types that have undergone extensive secondary cell wall (SCW) formation. These cell types are abundant in the stems of Arabidopsis, a well-studied model system for hardwood, the wood of eudicot plants. The main constituents of hardwood include cellulose, lignin, and xylan, the latter in the form of glucuronoxylan (GX). The binding of GX to cellulose in the eudicot SCW represents one of the best-understood molecular interactions within plant cell walls. The evenly spaced acetylation and 4-O-methyl glucuronic acid (MeGlcA) substitutions of the xylan polymer backbone facilitates binding in a linear two-fold screw conformation to the hydrophilic side of cellulose and signifies a high level of molecular specificity. However, the wider implications of GX–cellulose interactions for cellulose network formation and SCW architecture have remained less explored. In this study, we seek to expand our knowledge on this by characterizing the cellulose microfibril organization in three well-characterized GX mutants. The selected mutants display a range of GX deficiency from mild to severe, with findings indicating even the weakest mutant having significant perturbations of the cellulose network, as visualized by both scanning electron microscopy (SEM) and atomic force microscopy (AFM). We show by image analysis that microfibril width is increased by as much as three times in the severe mutants compared to the wild type and that the degree of directional dispersion of the fibrils is approximately doubled in all the three mutants. Further, we find that these changes correlate with both altered nanomechanical properties of the SCW, as observed by AFM, and with increases in enzymatic hydrolysis. Results from this study indicate the critical role that normal GX composition has on cellulose bundle formation and cellulose organization as a whole within the SCWs.

## Introduction

The global annual production of plant cell wall material has been estimated to be about 120–140 Gt of material (Pauly and Keegstra, 2008). The majority of this is secondary cell wall (SCW) material from land plants, such as trees (Bar-On et al., 2018; Zhong et al., 2019). Because of its massive abundance, the SCW material is of key interest as feedstock material for the growing bioeconomy, with knowledge of SCW formation, organization, and architecture being foundational to innovation in this area (Meents et al., 2018). While great strides have been made in understanding the cell wall composition and carbohydrate primary structure, our understanding of SCW formation during cell growth is still limited (Busse-Wicher et al., 2016a). A complete understanding of compositional and mechanistic interactions involved in SCW formation will provide directed avenues toward genetic and phenotypic modifications of plant biomass, stimulating innovation in plant biomass production and new applications, such as cellulose-based materials as substitutes for petroleum-derived products.

While the main role of primary cell walls is to control cell elongation and determine cell shape, SCWs are deposited after cell growth, and typically impart mechanical strength to the cell (Cosgrove and Jarvis, 2012). Characteristic cell types with SCWs exist in xylem tissues and sclerenchyma fiber cells (Meents et al., 2018). The SCWs in higher plants consist of ~40–55% cellulose, 20–30% hemicellulose, and 20–30% lignin (Pauly and Keegstra, 2008). As lignin is introduced later during wall formation, the initial synthesis and assembly of the SCW can be approximated as a two-component system consisting of cellulose and hemicellulose (Meents et al., 2018).

Cellulose microfibrils often appear in aggregate, or as bundles, in both the primary and SCWs. The bundles constitute a key architectural feature within the cell walls and range in size depending on the cell type and wall layer, e.g., SCW S1 layer vs. S2 layer (Donaldson, 2007; Cosgrove, 2014; Ding et al., 2014; Zhang et al., 2016). Cellulose bundles have been observed to be 10–30 nm in poplar and Arabidopsis SCW (Donaldson, 2007; Lyczakowski et al., 2019), 20–55 nm in spruce SCW (Donaldson, 2007; Jarvis, 2018; Lyczakowski et al., 2019), and from 5 to 30 nm in maize (Song et al., 2020).

Cellulose is produced at the plasma membrane by the cellulose synthase complex (CSC) in the form of elementary microfibrils and extruded into the apoplast between the plasma membrane and the inner side of the growing cell wall. Live cell imaging of CSCs during SCW formation indicates that clusters of CSCs moving in concert contribute to the assembly of cellulose bundles (Li et al., 2016). The CSC movement in the plasma membrane is guided from the cytosolic side by cortical microtubules (Lampugnani et al., 2019), but CSCs may also move autonomously, i.e., independent of microtubules, and maintain aligned trajectories. The latter presumably involves coalescence between the nascent microfibril of the CSC and *in muro* cellulose (Chan and Coen, 2020). Preparations of pure cellulose, such as nanocellulose and bacterial cellulose, self-organize in hierarchical fibrillary structures, which bear resemblance to native cellulose bundling in the cell wall (Martínez-Sanz et al., 2015). Hence, microfibrils frequently coalesce and form bundles as a part of cell wall assembly in a process that appears to be partly microtubule guided and partly autonomous.

The hemicellulose composition of the SCW is distinct among gymnosperms, monocots, and eudicots. This suggests that, while the SCWs between these three groups of plants share common features, some unique assembly and architectural principles are at play in each of these types of SCWs (Busse-Wicher et al., 2016b; Tryfona et al., 2019). The hemicellulose component of eudicot SCWs consists mainly of a single type of hemicellulose with a well-defined primary structure, i.e., glucuronoxylan (GX) (Smith et al., 2017). This compositional simplicity makes the SCW formation in eudicots, such as Arabidopsis, an attractive experimental system.

The primary structure of GX from Arabidopsis consists of a linear backbone comprised of xylose (Xyl) in 1,4-β-_D_-xylosidic linkages substituted with glucuronic acid (GlcA) or 4-O-methyl glucuronic acid (MeGlcA) at the Xyl*O*-2-position and with acetic acid at the Xyl*O*-2- and *O*-3-positions (Smith et al., 2017). In approximately half of the xylan molecules, a specific structure of β-_D_-Xyl-(1 → 4)-β-_D_-Xyl-(1 → 3)-α-_L_-Rha-(1 → 2)-α-_D_-GalA-(1 → 4)-_D_-Xyl exists in the reducing end (Pena et al., 2007; York and O'Neill, 2008). Notably, both [Me]GlcA substitutions, acetyl substitutions, and backbone length are nonrandom features of the GX molecule. Acetylation occurs on the Xyl residue approximately at every second (Busse-Wicher et al., 2014; Chong et al., 2014) and [Me]GlcA occurs in two distinct domains, major and minor, probably within the same GX molecule. The major domain has even number of Xyl residues in [Me]GlcA spacing, while the minor has no distinction between the even and odd spacing (Bromley et al., 2013). On average, [Me]GlcA substitution degree is ~12% (Brown et al., 2007). The GX backbone length, referred to as degree of polymerization (DP), is on average ~90 Xyl residues and forms a narrow-size distribution (Pena et al., 2007).

The highly non-random GX molecule is reflected by a complex synthesis pathway (Smith et al., 2017). Approximately, 20 genes have been implicated in GX synthesis and may be divided into three groups, namely backbone polymerization, DP regulation, and backbone decoration. The GX backbone formation is performed by what appears to be a protein complex consisting of the proteins, IRREGULAR XYLEM 9 (IRX9), IRX10, and IRX14 (Brown et al., 2005, 2007, 2009; Wu et al., 2009, 2010; Lee et al., 2010; Zeng et al., 2016; Smith et al., 2017). The 1,4-β-_D_-xylosidic linkage in the backbone is performed by IRX10 and its close homolog, XYLAN SYNTHASE 1 (XSY1) (Jensen et al., 2014; Urbanowicz et al., 2014), while enzymatic activity is not required for IRX9 function (Ren et al., 2014). Correct GX DP distribution involve the genes, such as *IRX7, IRX8*, and *PARVUS*, which ensure a narrow GX DP distribution (Lee et al., 2007; Pena et al., 2007), and *IRX15* and *IRX15-*L, which ensure correct GX DP average (Brown et al., 2011; Jensen et al., 2011). Notably, *IRX15* and *IRX15-*L do not affect the width of the GX DP distribution. Backbone decoration involves acetyl-, methyl-, and glucuronosyl-transferase activity and genes for each of these have been identified and characterized in several species (Rennie et al., 2012; Urbanowicz et al., 2012, 2014).

B-1,4-xylan is thought to have first evolved in charophyte algae where the pathway is significantly simpler than in higher plants, possibly limited to *KfIRX10* and a few decorating activities in *Klebsormidium flaccidum* (Jensen et al., 2018). Structural characterization of xylan and analysis for gene orthologs suggest that features, such as [Me]GlcA substitution (Kulkarni et al., 2012), acetylation on Xyl residue at every second (Haghighat et al., 2016) and reducing end group (Kulkarni et al., 2012) are later innovations in the evolution. Orthologs of *IRX9* and *IRX14* also appear to be required for xylan biosynthesis in the lower plants, such as *Selaginella moellendorffii* and *Physcomitrella patens* but appears not to be required for xylan biosynthesis in the mucilaginous layers of *Plantago ovata*, which is a eudicot (Jensen et al., 2014; Haghighat et al., 2016). It therefore appears that GX structure and synthesis pathway in dicot SCWs is both highly evolved and specialized. Accordingly, it suggests that SCW GX exerts an equally highly evolved and specialized function.

Molecular simulations, direct NMR evidence *in planta*, and elimination of even substitution pattern *in vivo* show that GX noncovalently binds to the hydrophilic side of cellulose in a two-fold helical screw and that the even substitution of GX is critical for a closely associated binding (Busse-Wicher et al., 2014; Simmons et al., 2016; Grantham et al., 2017). The GX may also bind to the hydrophobic side of cellulose, although in a less-defined manner (Busse-Wicher et al., 2016a). The major and minor domains of the GX molecule likely have different properties for interaction with cell wall components (Bromley et al., 2013).

From current scientific understanding, multiple possibilities exist on how GX, cellulose, and lignin interact, with an incomplete understanding of the role of GX in the SCW assembly (Busse-Wicher et al., 2016b; Simmons et al., 2016). The GX binding to cellulose may prevent fibril coalescence by steric hindrance (Grantham et al., 2017) or by inter-fibril repulsion based on electrostatic repulsion (Haigler et al., 1982; Reis and Vian, 2004; Yang et al., 2020). On the other hand, GX is long enough to crosslink fibrils (Busse-Wicher et al., 2016a), antiparallel xylan chains may dimerize through [Me]GlcA-mediated Ca^2+^-bridges (Pereira et al., 2017), and the presence of a xylan-transglycosidase in poplar SCW (Derba-Maceluch et al., 2015) may suggest that crosslinking GX molecules play a role in the SCW architecture. The GX may also serve a function by mediating lignin–cellulose interactions. The GX is more hydrophobic than cellulose and may act as a compatibilizer between the cellulose and the hydrophobic lignin matrix by binding to the hydrophilic side of the cellulose (Busse-Wicher et al., 2014). Further, circumstantial evidence suggests that GX-lignin crosslinks exist and that these could serve as bridges between the cellulose network and the lignin network (Terrett and Dupree, 2019).

Observations in GX mutants show evidence of altered cellulose organization and SCW architecture. Microscopy using cryopreservation shows that bundle diameter is significantly decreased in *irx9* and *irx10* (Lyczakowski et al., 2019) and transmission electron microscopy shows that the inner lining of the SCW is irregular in *irx9* and *irx15 irx15-L* (Pena et al., 2007; Jensen et al., 2011). However, more characterizations are needed if we are to gain a better understanding of these observations. In this study, we focus on the consequences of GX deficiency for cellulose organization and cell wall architecture in the SCW of Arabidopsis. We find increased cellulose bundling and increased orientational dispersion under high, medium, and weak GX deficiency. Our studies, therefore, suggest that GX is critical for correct cellulose network formation in Arabidopsis SCWs and provides insight on the implications of approaching xylan modification for improving biomass characteristics and for optimizing the production of bio-derived materials.

## Materials and Methods

### Arabidopsis Genotypes and Growth Conditions

The used plant genotypes included Arabidopsis wild type (*Col-0*) and T-DNA insertion mutants, *irx9* [Salk_057033; (Pena et al., 2007)], *irx10* (Salk_055673; Brown et al., 2005), *irx15 irx15-L* (GK_735E12, FLAG_532A08; Brown et al., 2011; Jensen et al., 2011), and *irx15 irx15-LIRX15-L* (line 1 in Jensen et al., 2011). The *IRX15-L* transgene in the latter line is driven by its own promoter. Seeds were planted in wet peat pellets and cold treated for 48 h at 4°C, then transferred to a growth chamber. Light/dark period was 16/8 h at a light intensity of 150 μE with light/dark temperature set at 23/20°C. Humidity was not controlled. After 3 weeks in the peat, pellet plants and pellets were transferred to individual pots measuring 8 × 8 × 12 cm with SureMix soil (Surefill, http://www.surefill.com/) and 3–4 plants per pot. Stems were harvested postsenesce and lyophilized to moisture content of 5% (g H_2_O/g biomass). Samples postmilling were stored in air-tight containers for further use. Particle size reduction for wide angle X-ray scattering (WAXS) and enzymatic hydrolysis was performed using a Wiley Mini-Mill (Thomas Scientific, Swedesboro, NJ) with a 30-mesh screen.

### Cell Wall Composition Analysis

Cell wall lignocellulosic material was isolated following the outlined extraction and destarching procedure (Foster et al., 2010b) using three sequential washes of 70% ethanol, 1:1 methanol-chloroform, and acetone to obtain alcohol insoluble residue (AIR). The AIR was destarched using 50 μg amylase/mL H_2_O (*Bacillus* species, Sigma-Aldrich, St. Louis, MO) and 18.7 units of pullinase (Bacillus acidopullulyticus, Sigma-Aldrich, St. Louis, MO) in a 0.01% sodium azide solution, with rotary mixing at 37°C overnight.

Non-cellulosic neutral monosaccharide content of the wall matrix polysaccharides was obtained by treating destarched AIR with trifluoracetic acid (TFA) followed by derivatization using the alditol acetate method with minor changes (Foster et al., 2010b). Crystalline cellulose was isolated following sequential hydrolysis of non-cellulosic polysaccharides with TFA, hydrolysis of non-crystalline cellulose using the Updegraff reagent followed by washing with water and acetone. The crystalline cellulose pellet was next hydrolyzed using sulfuric acid followed by quantification of the generated monosaccharides using the anthrone color metric assay (Foster et al., 2010b). Acetyl bromide soluble lignin (ABSL) was determined as described previously (Foster et al., 2010a). Samples were performed in technical triplicate and biological quadruplicate (*n* = 12), unless otherwise specified.

### Glycome Profiling

Previously-milled stem samples were ball-milled with a TissueLyser II (Qiagen Inc., Germantown, MD, USA) in preparation for sequential extraction. The sequential cell wall extractions of extractive-free Arabidopsis stems using oxalate, carbonate, 1 M KOH, 4 M KOH, acid chlorite delignification, and postchlorite extraction with 4 M KOH followed by glycome profiling of these extracts were carried out as described previously (Pattathil et al., 2012). Plant glycan-directed monoclonal antibodies (mAbs) were from laboratory stocks (CCRC, JIM, and MAC series) available at the Complex Carbohydrate Research Center (available through CarboSource Services; http://www.carbosource.net) or were obtained from BioSupplies (Australia) (BG1, LAMP). A description of the mAbs used in this study can be found in the >Supplementary Material of our prior work (Li et al., 2014).

### Scanning Electron Microscopy

Fresh nine-week stems were cut horizontally with a razor blade and fixed at 4°C for 1–2 h in 4% glutaraldehyde buffered with 0.1 M sodium phosphate at pH 7.4. Following a brief rinse in the buffer, the samples were dehydrated in an ethanol series (25, 50, 75, and 95%) for 15 min at each gradation and with three 15 min changes in 100% ethanol. Samples were critical point dried in a Leica Microsystems model EM CPD300 critical point dryer (Leica Microsystems, Vienna, Austria) using carbon dioxide as the transitional fluid. Samples were mounted on aluminum stubs using high vacuum carbon tabs (SPI Supplies, West Chester, PA, USA) and System Three Quick Cure 5 epoxy glue (System Three Resins, Inc. Auburn, WA, USA). Samples were coated with osmium (~10 nm thickness) in an NEOC-AT osmium chemical vapor deposition (CVD) coater (Meiwafosis Co., Ltd., Osaka, Japan), and examined in a JEOL 7500F (field emission emitter) SEM (JEOL Ltd., Tokyo, Japan) at an angle of ~70° between the longitudinal direction of the stem and the plane of image acquisition. Samples were prepared using three biological stems for each sample, and the images were acquired in triplicate (*n* = 3) at 200×, 14,000×, and 22,000× magnification.

### Atomic Force Microscopy

Air-dried 9-week old stem samples were selected from the first internode from the bottom for analysis. Longitudinal samples were prepared by hand-cutting using a platinum tipped razor blade in a petri dish, and delignified using a solution of 0.1 N HCl with 10% NaClO_2_ at 1% (w/v) biomass loading. Delignification occurred overnight at room temperature. After delignification, the samples were thoroughly washed with double-distilled H_2_O until the pH becomes neutral. Samples were transferred in a wet condition to a clean mica surface and were allowed to air dry at room temperature.

Image collection was performed using a Bruker Dimension FastScan (Bruker, Camarillo, CA, USA) equipped with an Acoustic and Vibration Isolation Enclosure, FastScan Scanner, Ultra-Stable High-Resonance Microscope Base, and a Nanoscope V Stage Controller and HV Amplifier. Scanning was performed using the PeakForce Mapping mode in air, with SCANASYST-AIR probes (Bruker, Camarillo, CA, USA). Tips were made of silicon nitride with a nominal tip radius of 2 nm, a resonant frequency of 70 kHz, and a spring constant of 0.4 N/m, which was appropriate for the range of modulus tested in this study.

Atomic force microscopy (AFM) operation and image preprocessing were performed in Nanoscope Analysis V1.5. All images were taken at 512 × 512 pixels, with four different scan sizes (2.5, 1.0, 0.5, and 0.25 μm) for each scan area, and at least three different scan areas measured for each sample (*n* = 3). All images were fitted with a third-order flatten prior to analysis to center data, remove tilt, and to remove the bow caused by an uneven cell surface. Image roughness, fibril width measurements, and height distribution profiles were all calculated in NanoScope Analysis V1.5 software using the Roughness, Section, and Particle Analysis tools, respectively. For fibril width analysis, AFM images in the 2.5 × 2.5 μm magnification were selected and five well-resolved features were identified in each picture and their width were measured using ImageJ. For microfibril orientation and dispersion analysis, three AFM images were analyzed for each genotype by using Fiji ImageJ (https://fiji.sc/) Directionality analysis based upon local gradient orientation on grayscale images.

Nanomechanical properties, including elastic modulus, adhesion, deformation, and dissipation, were standardized to the same z-axis properties for direct comparison of image intensities. Elastic modulus is calculated based upon the Derjaguin-Muller-Toporov model (Derjaguin et al., 1975). Deformation represents maximum penetration depth during tip-cell wall contact and can be related to the elastic modulus (Zhang et al., 2016). The adhesion force represents the absolute value of the negative force during tip release from the surface (Su et al., 2011). Dissipation is calculated by integrating the area between the extension and retraction curves and represents the difference in energy imparted from the AFM tip to the sample (Landoulsi and Dupres, 2013).

### Wide-Angle X-ray Scattering

X-ray diffraction (XRD) measurements were performed on a Bruker Davinci Diffractometer system (Bruker, Camarillo, CA, USA). The diffracted intensity of Cu Kα radiation (with Nickel filter; λ = 1.5418 Å; 40 kV and 40 mA) was measured in a 2 Theta range between 5 and 50° with Global Mirror method (Primary optics: 1.2 mm). The detector is in LYNXEYE ID mode and with a 3 mm slit. The milled sample mass for XRD measurement was about 1.5 g of total lower stem for each run. Sample collection was performed for 5 h total time at 0.0128° step sizes with 10 s of exposure at each step. Samples were performed in biological quadruplicate (*n* = 4). Sequential extraction of cell wall components was performed using the same methodology as described previously on extractive-free biomass (Li et al., 2014). Relative crystallinity index (RCI) was determined following the peak difference method and peak area methods outlined (Park et al., 2010) using Matlab v2012a (Mathworks, Natick, MA) for peak fitting with Gaussian distributions for crystalline and amorphous peak contributions.

### Enzymatic Hydrolysis

Enzymatic hydrolysis was performed at 1.0% solids loading (g alcohol insoluble residue (AIR) biomass/g solution) in 1.5 ml Posi-Click Tubes (Denville Scientific, Holliston, MA). Enzyme solutions were made at 30 mg protein/g glucan content using CTec3 and HTec3 (Novozymes A/S, Bagsværd, Denmark), with a CTec3:HTec3 ratio of 2:1 based on protein. Enzyme protein content was determined by the Bradford assay as reported in our prior work (Williams et al., 2017). A buffer solution of 50 mM citric acid (pH 5.20) was used to maintain pH, and the samples were incubated at 50°C with horizontal mixing at 180 rpm for 48 h. Samples were centrifuged at 13,000 × g for 3 min postincubation and filtered using 22 μm mixed cellulose-ester filters (EMD Millipore, Billerica, MA, USA). Samples were quantified on the HPLC (Agilent 1100 Series) with an Aminex HPX-87H column (Bio-Rad, Hercules, CA, USA) with a mobile phase of 5 mM H_2_SO_4_. Glucose (Glc) yield was determined based upon quantified Glc observed compared to total AIR cell wall glucan available (as Glc) including non-cellulosic glucan, while xylose (Xyl) yield was determined based upon the total AIR cell wall xylan available (as Xyl). Samples were performed in technical triplicate of biological duplicates (*n* = 6).

## Results

### Three Arabidopsis Mutants With Altered Xylan Biosynthesis

To understand the effect of glucuronoxylan (GX) on cellulose organization and secondary cell wall (SCW) architecture, we selected three well-characterized Arabidopsis loss-of-function mutants for this study, namely *irx9, irx10*, and *irx15 irx15-L*. The mutants were selected to have comparable impacts on GX synthesis while representing a progression in severity. As demonstrated in the prior literature and summarized in Table 1, the *irx9* GX deficient phenotype is the strongest, with severe reductions in crystalline cellulose, lignin, and xylose (Xyl) content relative to wild type along with reductions in GX DP by 70%. The *irx10* mutant was selected as the weakest of the three, showing no change in cellulose content, a 12–20% reduction in Xyl and with GX degree of polymerization (DP) reduced by ~39%. The *irx15 irx15-L* double mutant was selected as an intermediate severity mutant compared to *irx9* and *irx10*, displaying a 26% reduction in Xyl content, a 30%reduction in cellulose content, and a 50% reduction of GX DP relative to wild type (Brown et al., 2011; Jensen et al., 2011). The GlcA/ MeGlcA ratio is also shifted strongly toward 4-O-methyl glucuronic acid (MeGlcA) in all three mutants. Other aspects of GX primary structure, such as the extent of [Me]GlcA substitution and reducing end group occurrence are not affected in the selected mutants (Pena et al., 2007; Brown et al., 2009, 2011; Wu et al., 2009; Jensen et al., 2011).

**Table 1:** Three mutants deficient in glucuronoxylan (GX) biosynthesis.

**Mutants**	***irx9***	***irx10***	***irx15 irx15-L***
Irregular xylem (IRX) phenotype	Strong	Mild	Weak
Growth phenotype	Dwarfed	Normal	Normal
Cell wall thickness	Reduced and irregular	Reduced	Reduced and irregular
Crystalline cellulose content, previous studies	60%	100%	70%
Crystalline cellulose content, this study	90%^a^	105%^a^	100%
Lignin content, previous studies	24–50%	ND	ND
Lignin content, this study	65%^a^	100%	91%^a^
Xylose content, previous studies	40–50%	80–88%	74%
Xylose content, this study	61%^a^	100%	85%^a^
Xylan DP	30%	61–90%	50%
DP distribution	Narrow	ND	Narrow
GlcA/(Me)GlcA ratio	Dramatic reduction	Intermediate to dramatic reduction	Intermediate to dramatic reduction
[Me]GlcA substitution	Unaltered	Unaltered	Unaltered
Reducing end group	Unaltered	Unaltered	Unaltered
Literature reference	Brown et al., 2005; Pena et al., 2007; Wu et al., 2010; Lee et al., 2014	Brown et al., 2005, 2009; Wu et al., 2009	Brown et al., 2011; Jensen et al., 2011

a *Statistically different from wild type (p < 0.05)*.

Differences in growth conditions and analysis protocols between previous studies employing *irx9, irx10*, and *irx15 irx15-L* mutants prevent direct comparisons of characterization results among these studies. To directly compare their cell wall composition and SCW structural features, the three GX mutants were grown in parallel in several batches and were characterized using a range of approaches. *Col-0* was included in the studies as wild type control. The *irx15 irx15-L* double mutant is a cross between T-DNA lines generated in different ecotypes, i.e., *Col-0* and wild types. We, therefore, included a complementation line from Jensen et al. (2011) displaying wild type level of the *IRX15-L* transgene, in this study designated as *irx15 irx15-L IRX15L*, to serve as an additional control for *irx15 irx15-L*.

The structural polysaccharides and lignin content were determined for extractive-free inflorescent stem material (Table 1, Supplementary Figure 1). The results show that the compositional phenotype of all three mutants is milder under our growth conditions compared to previous studies. Notably, *irx10* shows a minor increase in crystalline cellulose and no change in Xyl content compared to wild type, and *irx15 irx15-L* shows no reduction in crystalline cellulose compared to both wild type and *irx15 irx15-L IRX15L*. The *irx* phenotype of collapsed xylem vessels were also not observed for *irx10* (Supplementary Figure 1). The milder mutant phenotypes must be given extra care by watering and fertilizing the plants in order to promote maximal biomass production and ensure enough plant material for some of the experiments. For the same reason, the stem material was harvested late in the growth cycle toward senescence, but may also represent a difference in growth conditions compared to previous studies. The trend of the mutant phenotypes across the three mutants is however consistent with the previous studies.

**Figure 1:**
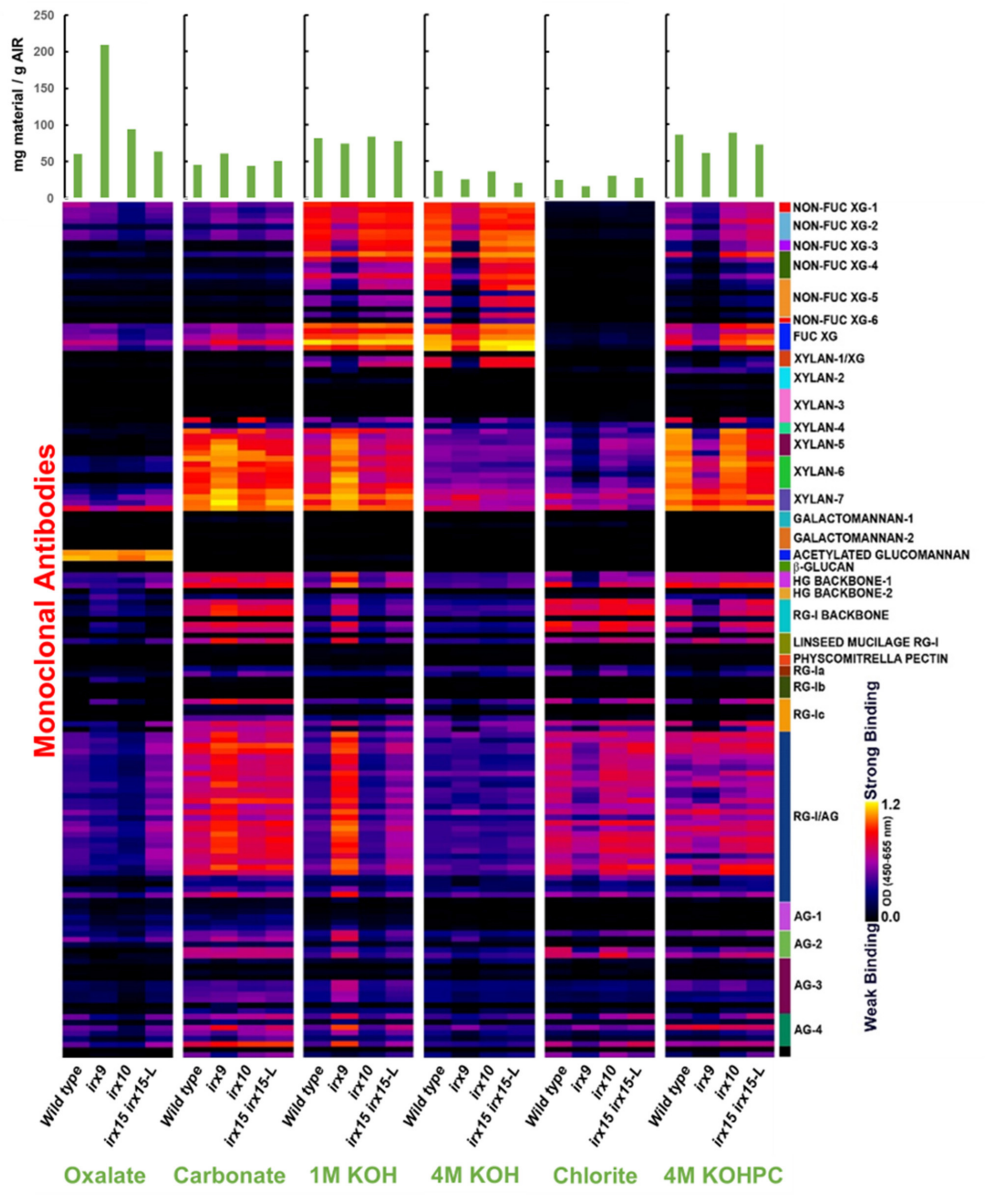
Glycome profile analysis of cell wall material of glucuronoxylan (GX) mutant stems. Cell wall material of wild type, *irx9, irx10*, and *irx15 irx15-L* inflorescence stems was extracted sequentially with increasing chemical severity. Quantification of the different cell wall carbohydrates by antibody binding is presented as a color gradient as depicted to the left, black (low) to yellow (high). Green bars on the top indicate the amount of carbohydrate recovered per gram of cell wall alcohol insoluble residue (AIR) for each extraction.

Glycome profiling was next employed to assess the changes in the extractability of the non-cellulosic cell wall polysaccharides associated with the *irx* mutations (Figure 1). The glycome profiling technique subjects the cell wall to increasingly harsh solvent extractions to selectively solubilize matrix polysaccharides, with the solubilized extracts subsequently interrogated against a panel of glycan-specific antibodies. The results can yield information about both the relative abundance of particular glycan epitopes as well as how strongly these glycans are associated with the cell wall (Li et al., 2014).

Results from glyome profiling demonstrate that changes in the abundance and extractability of several key glycan epitopes correlate directly with the severity of xylan deficiency. Epitopes associated with xylan, xyloglucan, homogalacturonan, RG-I, and type II arabinogalactan all show substantial changes in *irx9* and *irx15 irx15-L* compared to the wild type (Figure 1). Specifically, xyloglucan epitopes are reduced in the 1M and 4M KOH extractions with no concurrent increase of these epitopes in any of the other fractions. The epitopes for pectic polysaccharides and arabinogalactan display a similar pattern to the xylan epitopes by being more abundant in the carbonate and 1M KOH fractions, and less abundant in the successive extractions. This indicates that the pectic polysaccharides, arabinogalactan and xylans have increased extractability in the *irx9* and *irx15-L IRX15-L* lines, which is consistent with the previous reports (Pena et al., 2007; Brown et al., 2011; Jensen et al., 2011). Only subtle differences in epitope binding are observed between *irx10* and wild type. The severe reductions in GX, cellulose, xyloglucan, and lignin in *irx9* are attributed to the reduction in SCW thickness present in this mutant. The control line *irx15 irx15-L IRX15-L* was not included in the glycome profiling.

Together, both the results of compositional analyses and glycome profiling confirm the increasing differences in SCW formation in the three GX mutants as severe, medium, and weak for *irx9, irx15 irx15-L*, and *irx10*, respectively. Notably, *irx10* shows minimal impact on the overall SCW composition and extractability, while multiple changes occur in *irx9* and *irx15 irx5-L*.

### Cellulose Microfibril Organization Is Altered in Xylan Mutants

To investigate whether cellulose organization is altered by the differing levels of GX deficiency, we employed scanning electron microscopy (SEM) and atomic force microscopy (AFM) (Ding et al., 2014) to the inner and most recently deposited SCW surface. We chose stem interfascicular fiber cells as a model system as these produce a thick and uniform SCW. In order to make the irregularities of the inner side of the wall to stand out clearly, we recorded SEM images at a tilted angle.

**Figure 2:**
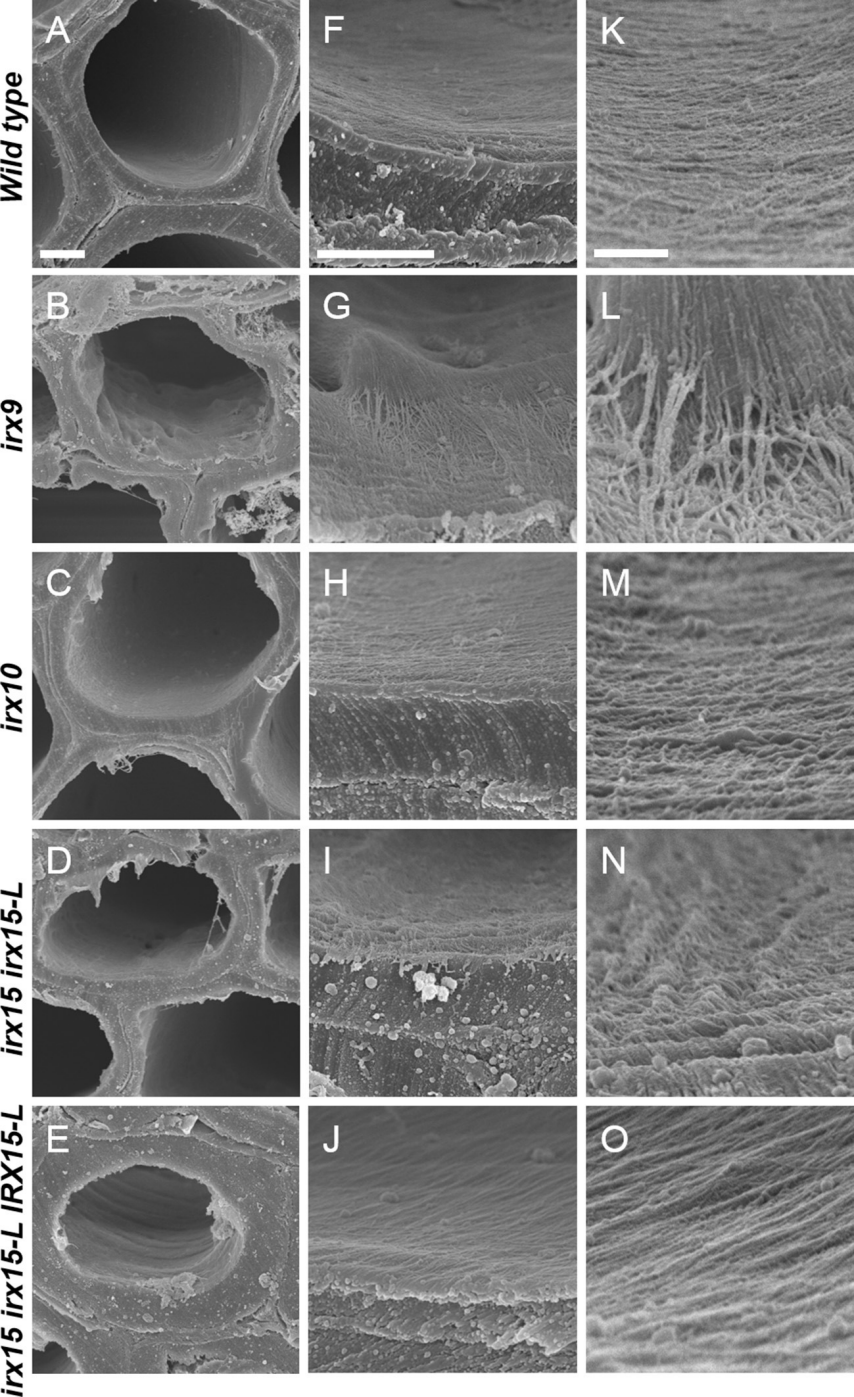
Ultrastructure of glucuronoxylan (GX) mutants observed by scanning electron microscopy (SEM). Scanning electron microscopy images of inner surface texture of interfascicular fiber cells in Arabidopsis. Scale bars are 2 μm for low magnification **(A–E)**, 1 μm for intermediate magnification **(F–J)**, and 200 nm for high magnification **(K–O)**.

Scanning electron microscopy (SEM) images of wild type cell walls reveal a smooth and even surface at the lowest magnification and a smooth and unidirectional fibrous patterning at medium and high magnification. The *irx15 irx15-L IRX15-L* control is indistinguishable from the wild type. In contrast, a range of malformed features are observed in the three GX mutants (Figure 2, Supplementary Figures 2–6). Malformations are discernable at both low and high magnification in *irx9* and *irx15 irx15-L*, while the changes are smaller in *irx10* but clearly discernable at the higher magnifications. A common feature observed in the GX mutants is that both individual fibrillary structures and overall patterning appear coarser compared to the wild type. The overall patterning in all mutants is affected by fibrillary structures diverging from the general direction. Also common in the GX mutants, is a wave pattern, resembling “rolling hills,” with a periodicity in the 100–200 nm range. The severity of these observations correlates clearly with the extent of GX deficiency in the individual mutant.

**Figure 3:**
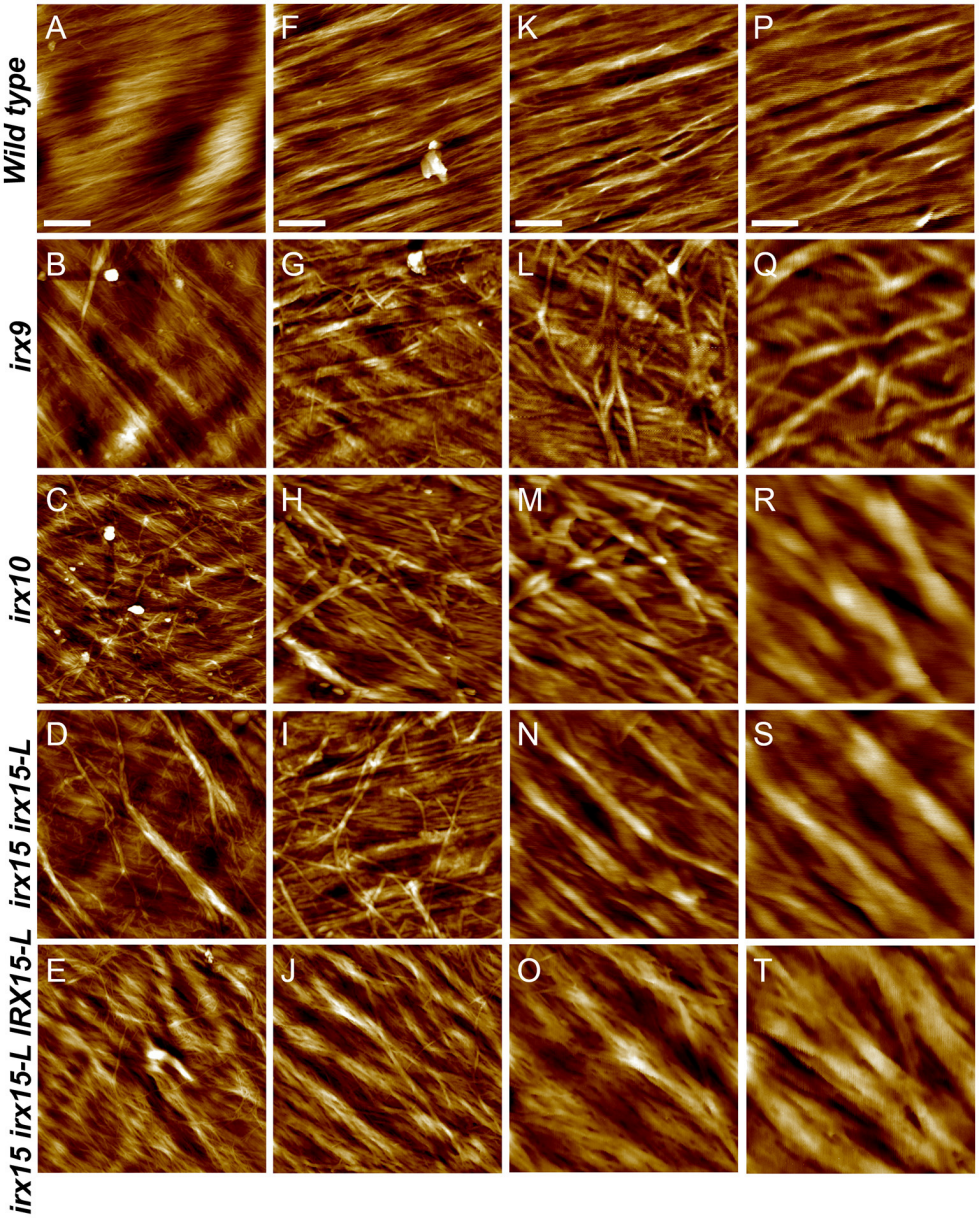
Ultrastructure of glucuronoxylan (GX) mutants by atomic force microscopy (AFM) height mapping. Atomic force microscopy images of inner surface texture of interfascicular fiber cells in Arabidopsis. Scale bars are 500 nm **(A–E)**, 200 nm **(F–J)**, 100 nm **(K–O)**, and 50 nm **(P–T)**.

**Figure 4:**
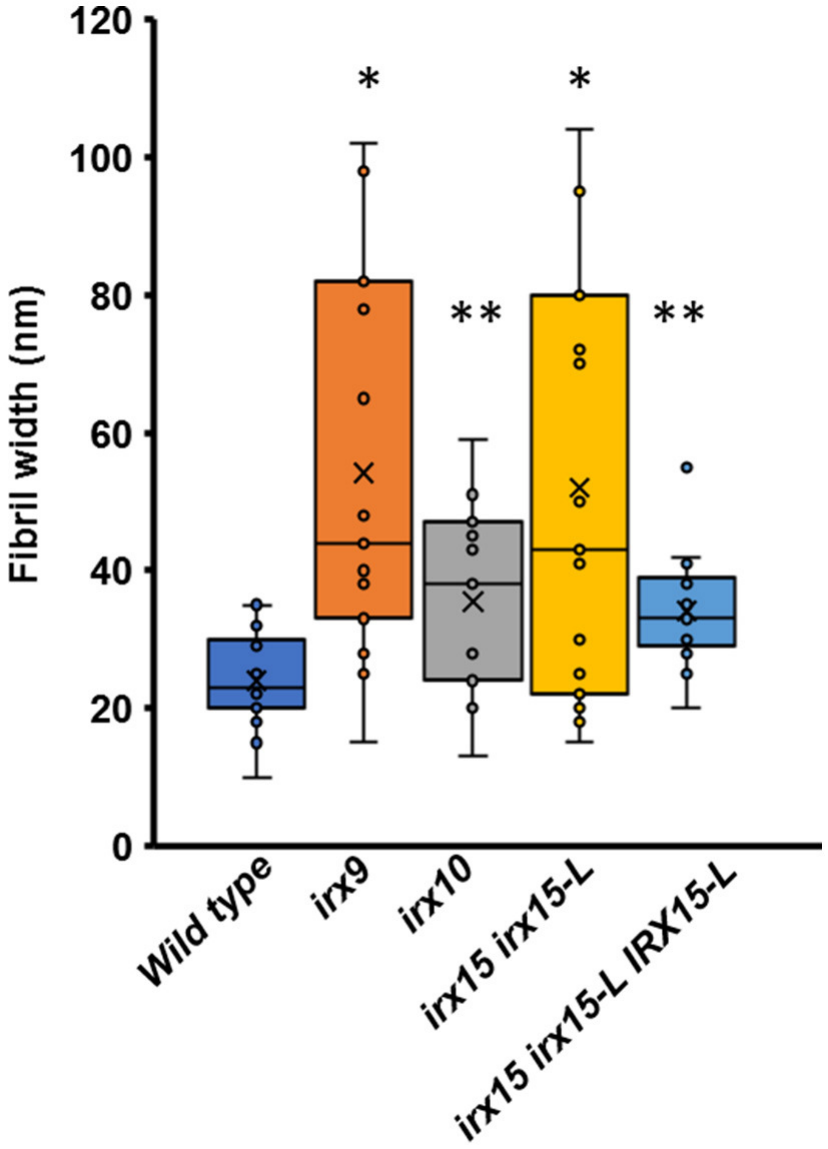
Fiber width of wild type and GX mutants using atomic force microscopy (AFM). Fibril widths were determined from well-resolved features in 2.5 × 2.5 μm image sizes, using five measurements for each of three individual images for each sample (*n* = 15). The symbol * indicates statistical significance compared to wild type (*t*-test, *p* < 0.05), and ** indicates statistical significance from both wild type and *irx9* and *irx15-15-L* mutants.

Atomic force microscopy (AFM) was next employed to further characterize the cell wall architecture at high magnification. In order to visualize the GX-cellulose microfibrils clearly, the samples were first subjected to mild delignification as reported in a prior work (Lacayo et al., 2010). Images of the inner SCW of wild type reveal a smooth surface of microfibrillar structures with uniform orientation (Figure 3, Supplementary Figures 3–6), which we hypothesize to be GX-cellulose microfibrils. Imaging below the 0.25 × 0.25 μm scale did not improve image resolution, given the roughness of the surface. Upon inspection, it is clear that smaller microfibrils come together in bundles and that these further come together in regional collections of more loosely defined agglomerates. These larger fibrillary agglomerations may extend for 200–300 nm or longer before branching, the branches often merging with other fibrillary agglomerations in the immediate vicinity. Crevasses appear in the weaving pattern side by side with the fibrillary agglomerations. These observations are not unlike those of the inner SCW in *Zinnia elegans* tracheary elements (Lacayo et al., 2010). In contrast, the GX mutants show a clear increase in fibrillary misalignment, agglomerate size, and crevasse width and appear distinctly disorganized compared to the wild type. Misalignment and increased microfibrillar bundling lead to increased surface roughness and cause the resolution at the highest magnification to decrease in the GX mutants. Bundles running transverse to the general direction appear in each of the three mutants and are clearly larger and more complex in *irx9* and *irx15 irx15-L*. The complementation line almost reverts the *irx15 irx15-L* phenotype to wild type. The fibrillar pattern in this line is wavier compared to the wild type, while crevasses are bigger and stray bundles occur frequently. However, the general pattern is more regular than the one found in *irx10*. Quantification of the width of the largest agglomerates in 2.5 × 2.5 μm images show that all the three GX mutants display larger agglomerates, with those of *irx15 irx15-L* and *irx9* being ~3times larger than the wild type (Figure 4). Agglomerate size in the complementation line is comparable to that of *irx10* and is significantly reduced compared to *irx15 irx15-L*.

**Figure 5:**
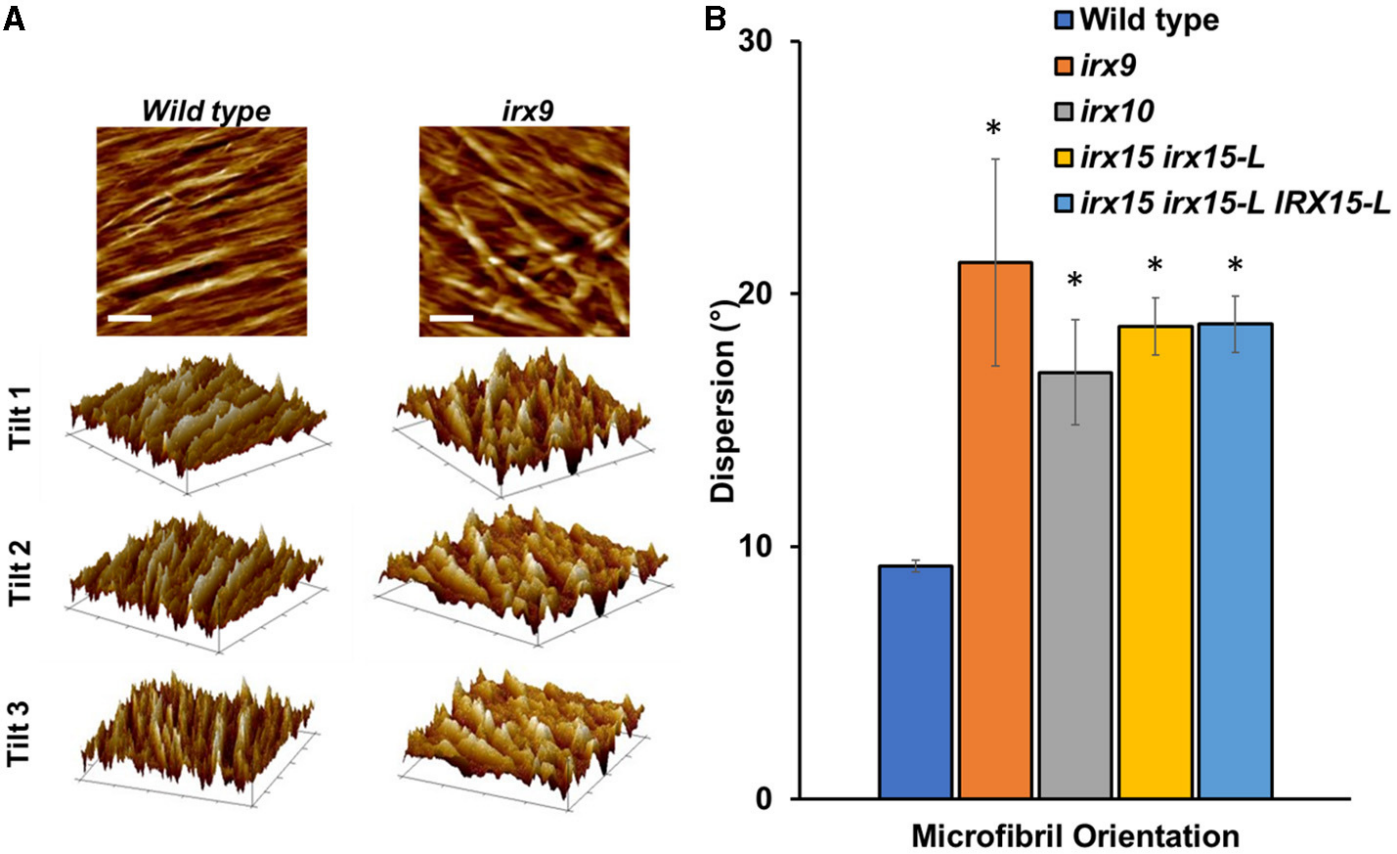
Fiber orientation and 3D topography of wild type and glucuronoxylan (GX) mutants using atomic force microscopy (AFM). **(A)** Height profile image (0.5 × 0.5 μm; scale bar = 100 nm) and its corresponding 3D surface projections at three different angles of wild type and *irx9*. The parallel striation observed in the wild type height images can be recognized as parallel ridges and groves in the 3D surface projections. This type of structural organization is strongly reduced in *irx9*. **(B)** Microfibril orientations measured by dispersion of fibers distributed throughout at the 1.0 x 1.0 μm scale. Standard deviations are shown (*n* = 3). * indicates statistical significance compared to wild type (*t*-test, *p* < 0.05).

We next sought to quantify bundle directionality and surface roughness by computational image analysis. Bundle orientation is a measure of directionality within an image, with dispersion measuring the deviation from the mean angle of orientation. Factors that may contribute to dispersion are bends, breaks, and out of plane features (Yan et al., 2017). Directionality is pronounced in the wild type with clear groves are visible by AFM surface topography graphs (Figure 5A) and a dispersion of ~10° (Figure 5B). Comparably, all the three GX mutants show significantly higher dispersion values (*p* < 0.05), with *irx9* showing the largest average deviation value of ~20° (Figure 5B). Dispersion remains high in the complementation line consistent with the occurrence of stray bundles and wavier patterns compared to the wild type.

Roughness quantifies irregularities in surface features from a mean plane, with larger irregularities corresponding to higher values for roughness. This value is dependent upon both the sample scale and measurement size (De Oliveira et al., 2012). Average roughness (R_a_) and root-mean-square roughness (R_q_) were used to quantify average image roughness and deviation from mean roughness, respectively. At the 0.5 × 0.5 μm scale, R_a_ and R_q_ values for *irx9, irx15 irx15-L* and complementation line are at least 50% higher compared to the wild type. However, both the properties exhibit large variation in these three lines such that these differences are statistically significant only in the complementation line (Table 2). The high R_a_ and R_q_ values most likely reflect the uneven and wavy surfaces rather than irregular microfibril organization. R_a_ and R_q_ values for *irx10* are closer to wild type and show lower variation such that the differences between *irx10* and wild type are statistically significant (*p* ≤ 0.05). Roughness analysis for the other three AFM magnifications shows similar trends, however with lower statistical confidence (Supplementary Table 1).

**Table 2:** Roughness analysis of secondary cell walls of wild type and glucuronoxylan (GX) mutants.

**Genotype**	**R^a^/nm^a^**	**R_q_/nm^b^**
Wild type	0.70 ± 0.02^b^	0.89 ± 0.03
*irx9*	1.09 ± 0.18 *p* = 0.08^c^	1.83 ± 0.73 *p* = 0.09
*irx10*	0.92 ± 0.05 p = 0.03	1.19 ± 0.04 p = 0.02
*irx15 irx15-L*	1.11 ± 0.19 *p* = 0.06	1.41 ± 0.27 *p* = 0.09
*irx15 irx15-L IRX15-L*	1.50 ± 0.33 *p* = 0.05	1.91 ± 0.40 *p* = 0.05

a *Roughness parameters for average roughness (R_a_) and root-mean-square roughness (R_q_) measured on atomic force microscopy (AFM) height images at 0.5 × 0.5 μm image*.

b *Standard deviations shown (n = 3)*.

c *T-test P-values compared to wild type*.

We conclude that there is an increase in the roughness of the inner SCW surface, caused by a decrease in unidirectionality and an increase in microfibril agglomeration for all the three GX mutants. The characteristics of the SEM images of coarser fibrillary patterning corroborate with the observations made by AFM. Notably, the severity of cellulose disorganization corresponds to the degree of GX deficiency by both SEM and AFM.

### Altered Cellulose Organization Affects Nanomechanical Properties

To correlate the cell wall architecture with mechanical properties, we recorded nanomechanical properties, by AFM, namely elastic modulus, adhesion, deformation, and dissipation, for each pixel of the surface topology scans, generating spatial distribution maps of each of these properties. Elastic modulus is a measure of the ratio of the force exerted on a material to resulting deformation and is, in the context of AFM, based upon contact deformation between the AFM tip and cellulose microfibril surface (Derjaguin et al., 1975). High modulus regions thereby represent stiffer surfaces and correspond to lower deformation distances. The adhesion force represents the absolute value of the negative force during tip release from the surface (Su et al., 2011), while dissipation is measured based upon the electrostatic discharge, and may be representative of electrochemical surface heterogeneity of soft matter (Garcia and Proksch, 2013).

**Figure 6:**
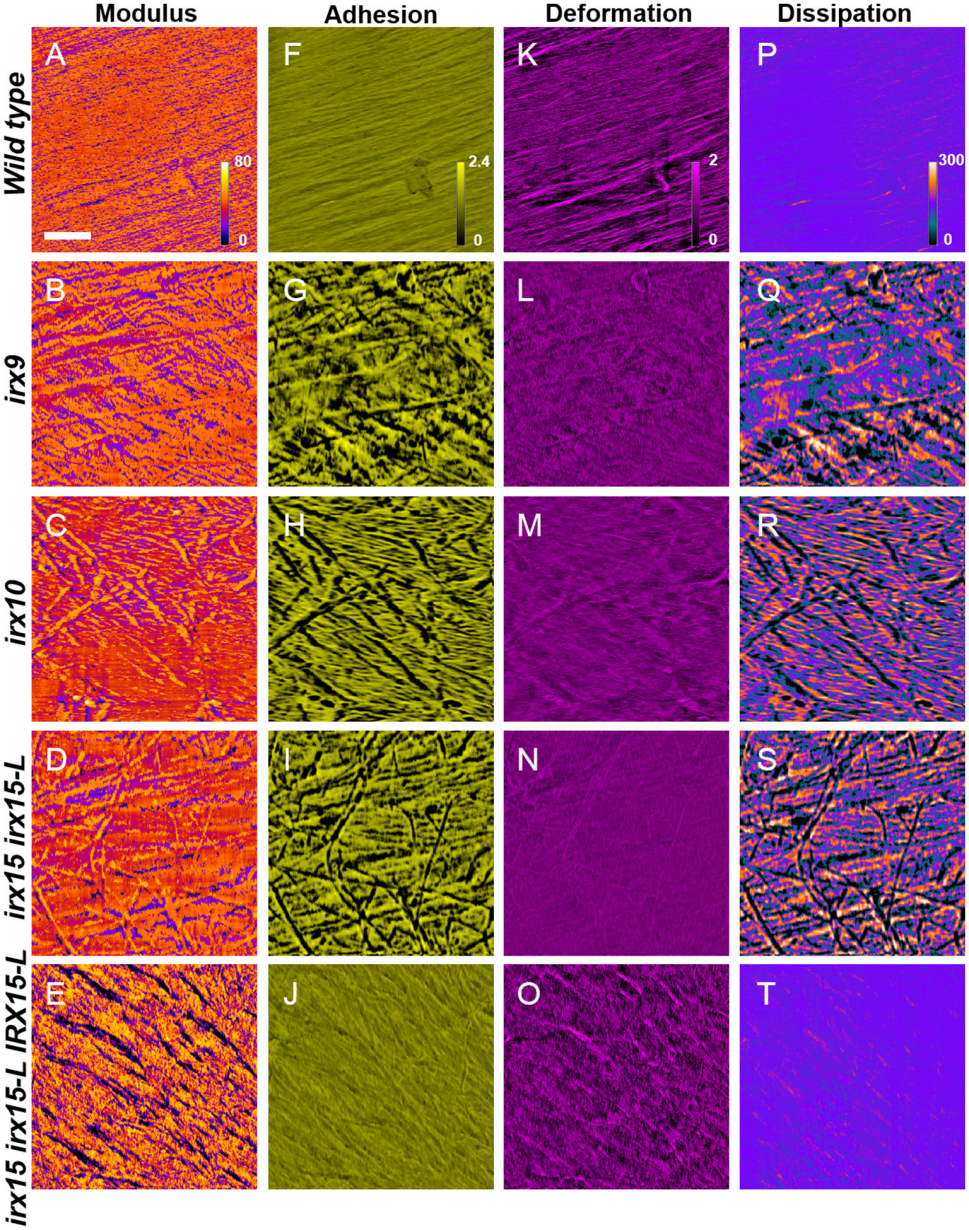
Nanomechanical imaging of glucuronoxylan (GX) mutants by atomic force microscopy (AFM). Nanomechanical mapping of modulus **(A–E)**, adhesion **(F–J)**, deformation **(K–O)**, and dissipation **(P–T)** of wild type and irx mutants. Scale bar represents 200 nm, with all images corresponding to a 1.0 × 1.0 μm image size.

Atomic force microscopy images revealed uniform features in the wild type for all the four properties, correlating a fine and evenly organized cellulose fibril network with uniform nanomechanical properties, even postdelignification (Figure 6). This is in contrast to the nanomechanical recordings of the GX mutants, which show large spatial variation in adhesion and dissipation and some changes in the modulus. The unevenness in the mechanical properties overlay with the disorganized cellulose network observed in the topology scans (Figures 3, 6).Thus, surface strength and electrostatic environment in the GX mutants is altered and correspond to the altered cellulose organization. The complementation line has adhesion and dissipation similar to the wild type, while the modulus is further increased in heterogeneity compared to the modulus of any of the three GX mutants. The images display what appear to be grooves that have low modulus, i.e., soft surface with high deformation. The groves are replicated in the deformation images. Hence, the complementation line reverts the electrostatic heterogeneity found in *irx15 irx15-L*, while the mechanical properties are increased in heterogeneity.

### Xylan Deficiency Leads to Altered Cellulose Crystallinity as Determined by X-ray Diffraction

**Figure 7:**
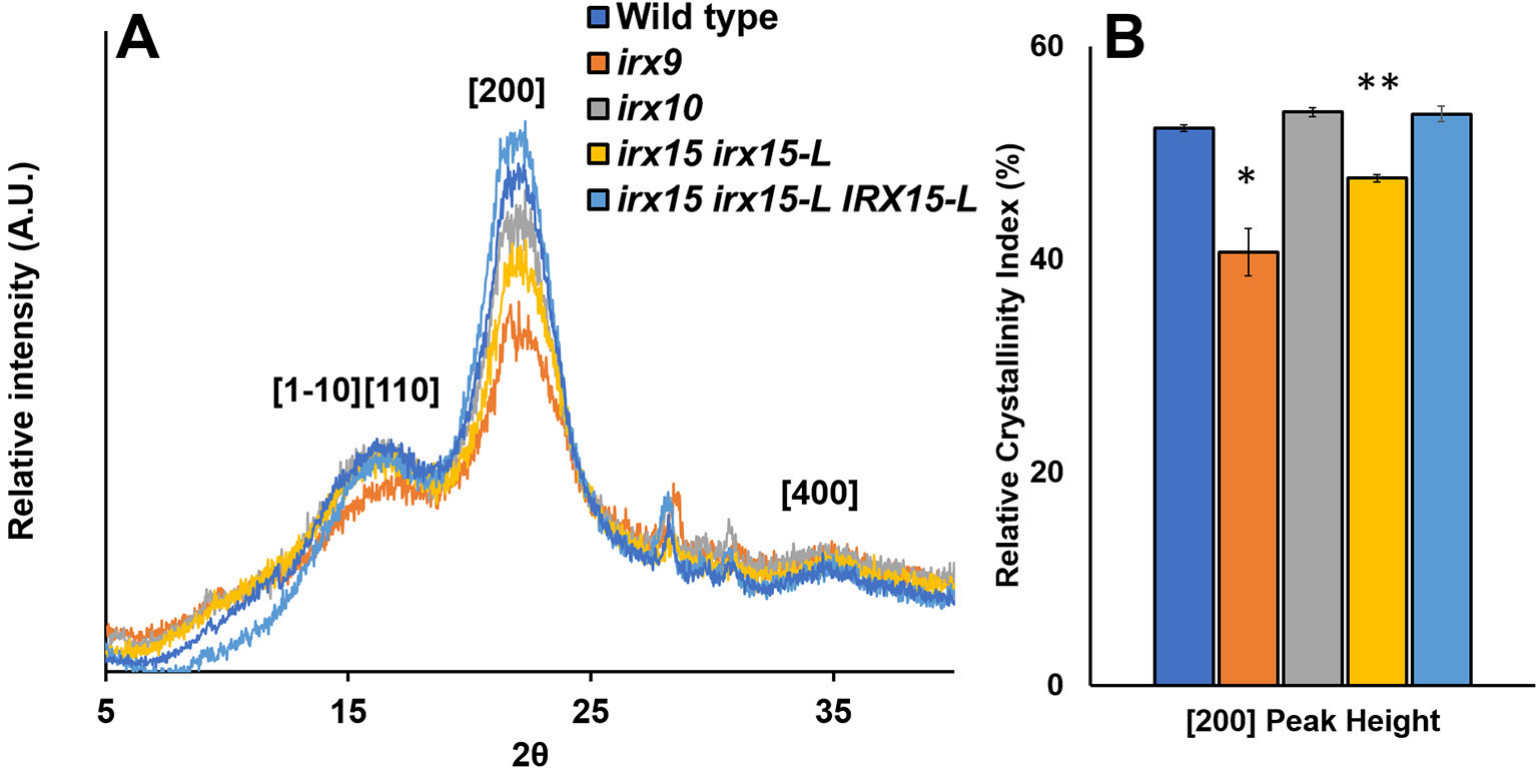
Wide-angle X-ray scattering (WAXS) diffraction pattern of stem cell wall material from wild type and glucuronoxylan (GX) mutants. **(A)** Representative wide-angle X-ray scattering **(**WAXS) diffraction patterns of wild type, *irx9, irx10*, and *irx15 irx15-L* stems showing the diffraction peaks for the [110], [1–10], [200], and [400] crystal faces. **(B)** Relative crystallinity indices of the [200] peak face calculated from the WAXS diffraction pattern, such as the ones in **(A)**. Standard deviations are shown (*n* = 4, biological replica). * indicates statistical significance compared to wild type (*t*-test, *p* < 0.05), and ** indicates statistical significance from wild type and *irx9*.

Wide-angle X-ray scattering (WAXS) is a non-destructive technique that has been applied as a tool for probing cellulose structure and crystallinity (Cheng et al., 2015). As each selected genotype shows clear changes in cellulose fibril organization, we tested to find if any of these changes corresponded to recurred microfibril crystallinity and could be quantified by WAXS. For *irx15 irx15-L*, and *irx9*, the [200] crystal face decreases relative to the [1–10] and [110] crystal faces. For *irx9* only, a reduction in the [1–10] and [110] crystal faces compared to the amorphous base line is also evident (Figure 7A). Quantification of relative crystallinity index (RCI) by the peak difference method (Supplementary Figure 7A; Park et al., 2010) reveals a lower calculated RCI of the [200] crystal face in *irx15 irx15-L* and *irx9* by 9 and 22%, respectively (Figure 7B). No statistical differences compared to wild type are evident for *irx10*. The RCI is reverted completely to wild type level in the complementation line. The RCI using the peak area method yields similar conclusions despite higher errors associated with the technique (Supplementary Figure 7B).

Reductions in RCI can be attributed to either a reduction in crystalline chains or an increase in crystallite size (Somerville, 2006; Taylor, 2008; Takahashi et al., 2009). Relative crystallite size of the [200] face determined using the Sherrer correlation (Patterson, 1939) was found for all samples to be ~4.25 nm on average (Supplementary Figure 7C), which is comparable to prior studies (Newman et al., 2013) and agrees with the mean elementary fibril width proposed in the cellulose 36-chain model (Ding et al., 2012). As a result, we hypothesize that changes in RCI are to be caused by changes in crystalline cellulose abundance rather than crystallite size. In the case of *irx9*, a 10% reduction in crystalline cellulose content measured by acid hydrolysis (Table 1) could account for the reduction in RCI; however, *irx15 irx15-L* shows no observed reduction in the crystalline cellulose content by chemical quantification. Hence, since neither crystallite size nor cellulose content is reduced in *irx15 irx15-L*, it suggests that the altered X-ray scattering properties of this genotype may be attributed to organizational changes in the cellulose network.

**Figure 8:**
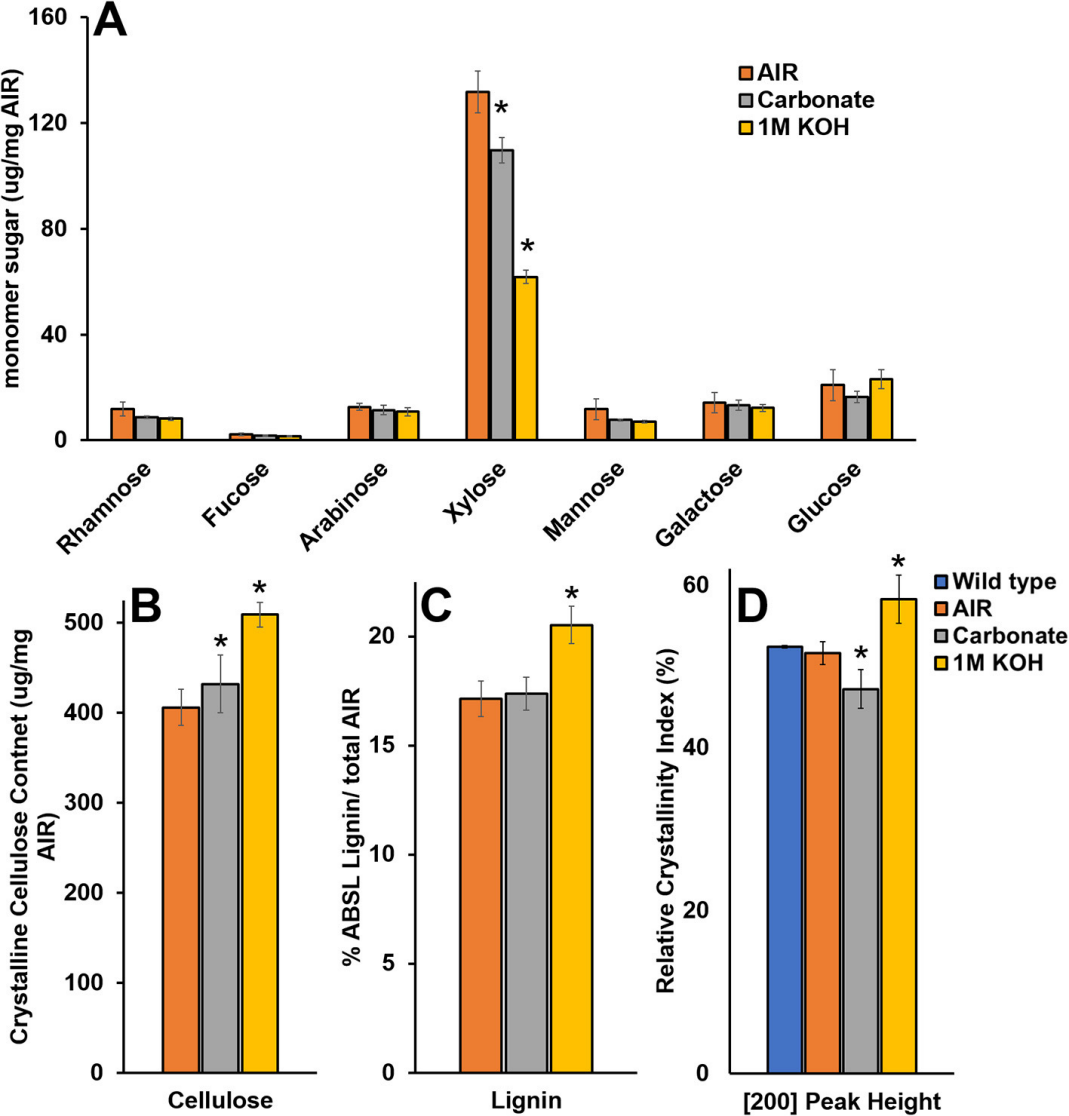
Chemical composition and wide-angle X-ray scattering (WAXS) relative crystallinity index of wild type cell wall material after chemical extraction. **(A–C)** Chemical composition analysis of wild type alcohol insoluble residue (AIR) and AIR sequentially extracted with carbonate and 1M KOH. **(D)** Relative crystallinity indices (RCIs) of the [200] peak face calculated from the WAXS diffraction patterns in wild type native, AIR, and sequentially extracted with carbonate and 1M KOH. Standard deviations are shown (*n* = 3, biological replicates. *Indicates statistical significance compared to wild type (*t*-test, *p* < 0.05).

To study the effect of cell wall material with reduced matrix polysaccharide content on WAXS under controlled conditions, we extracted wild type SCW material with carbonate and KOH and characterized the material by monosaccharide composition, crystalline cellulose content, lignin content, and WAXS RCI (Figure 8). Extraction using 1M KOH resulted in ~50% reduction in Xyl and significant increases in crystalline cellulose, lignin, and RCI. Extraction using carbonate resulted in ~15% reduction in Xyl, an increase in crystalline cellulose, no change in lignin, and a significant reduction in RCI. Hence, carbonate extraction mimics the *irx15 irx15-L* phenotype where crystalline cellulose is uncoupled from a reduced RCI. It is unlikely that carbonate extraction should change cellulose crystallite size. Thus, matrix polysaccharide extraction causes a lower RCI, which may be ascribed to organizational changes in the cellulose microfibril network.

### Altered Cellulose Organization Leads to Increase in Enzymatic Hydrolysis

**Figure 9:**
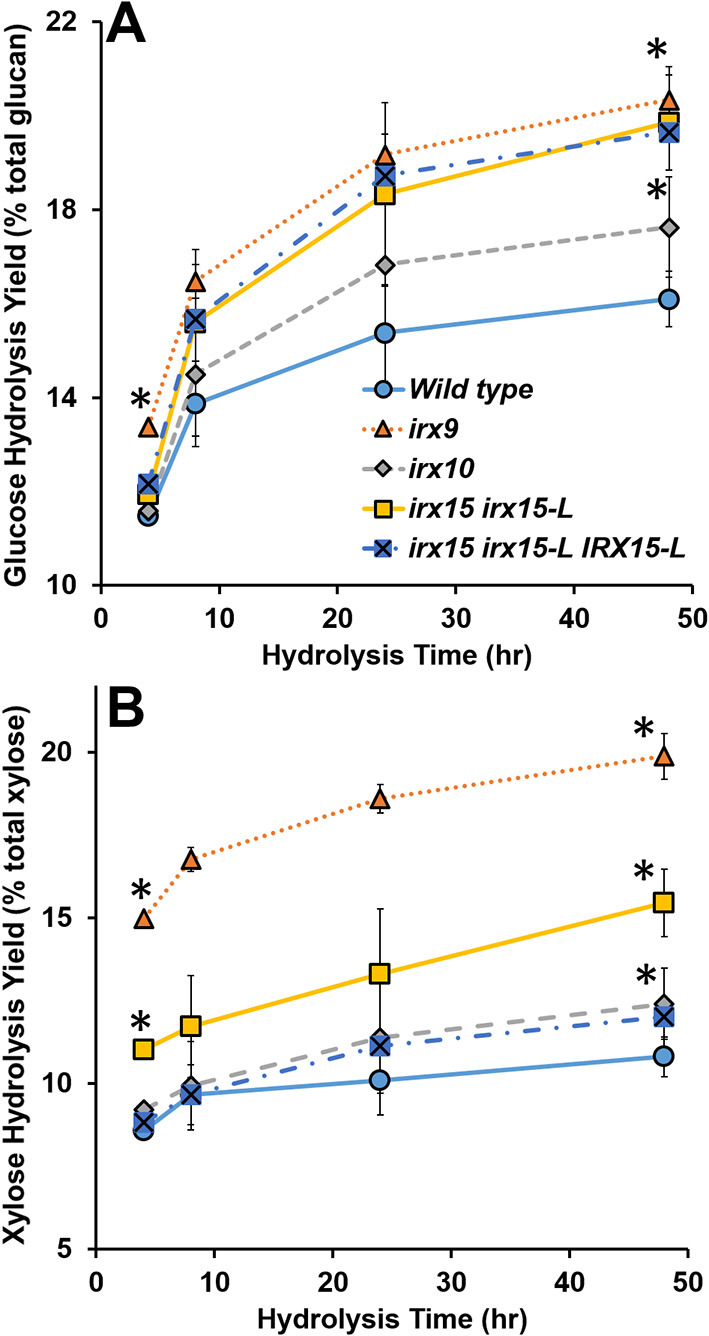
Enzymatic digestibility of cell wall material of glucuronoxylan (GX) mutant stems. Enzymatic hydrolysis monomeric sugar yields of glucose (Glc) **(A)** and xylose (Xyl) **(B)** of unpretreated alcohol insoluble residue (AIR) on the basis of % monomeric sugar observed per total available polysaccharide (g/g*100). Timepoints for the measurements were 4, 8, 24, and 48 h. Standard deviations are shown (*n* = 6, biological duplicate with technical triplicate). *Indicates statistical significance compared to wild type (*t*-test, *p* < 0.05).

Lastly, we considered enzymatic hydrolysis as a means to access changes in the SCW formation. We hypothesized that if the SCW structure is more porous, the SCW polysaccharides may hydrolyze more easily, particularly in case of no pretreatment. To test this, we conducted a hydrolysis time course experiment quantifying glucose (Glc) and xylose (Xyl) release. The results show that Glc and Xyl are indeed more easily released for all the three GX mutants compared to the wild type (Figure 9). As per the results from the other analyses, *irx9* and *irx15 irx15-L* showed the largest change compared to the wild type. Notably, saccharification of the complementation is unchanged compared to *irx15 irx15-L*. The increase in Glc release points to a significant change in cellulose organization and so the enzymatic hydrolysis corporates our findings by SEM and AFM that the cellulose organization in the GX mutants is significantly impacted.

## Discussion

### Glucuronoxylan Affects Fibril Agglomeration and Alignment

We demonstrate that glucuronoxylan (GX) is critical for uniform cellulose bundling and alignment in a dosage dependent manner, i.e., the stronger the GX deficiency, the stronger the impact on cellulose organization. First, Scanning electron microscopy (SEM) reveals uneven cellulose organization which correlates with the degree of GX deficiency. Secondly, atomic force microscopy (AFM) reveals increased cellulose bundling and misalignment in all the three GX mutants. Cell wall disorganization in *irx10* is particularly distinct by the AFM approach compared to SEM. The appearance of a smooth inner surface of cellulose bundles with uniform orientation in the wild type suggests that delignification, which is a part of sample preparation, did not result in large-scale rearrangements. This is consistent with our prior observations in secondary cell walls (SCWs) of maize (Ding et al., 2014) and is further supported by observing a good correspondence between the SEM and AFM data along with the fact that SEM sample preparation did not involve delignification. Third, each of the three mutants shows similar nanomechanical changes. The changes overlap with the altered cellulose organization and suggest that the electrostatic environment of the inner SCW surface is altered. Fourth, *irx15 irx15-L* shows altered cellulose organization by wide-angle X-ray scattering (WAXS). Though subtle differences exist between the WAXS spectra of *irx10* and wild type, the relative crystallinity index (RCI) calculations showed no statistical difference. The RCI of *irx9* is lower but this cannot be assigned to changes in cellulose organization exclusively because crystalline cellulose content is strongly reduced in this mutant. Fifth, increased release of both xylose (Xyl) and glucose (Glc) by enzymatic hydrolysis argue that significant changes in both GX and cellulose organization is present in the glucuronoxylan (GX) mutants.

We primarily attribute the changes observed in the three mutants to changes in GX degree of polymerization (DP) and total GX content. Other changes in GX primary structure in the three mutants are minor, such as reduced glucuronic acid (GlcA)/4-O-methyl glucuronic acid (Me)GlcA ratio, or unchanged, such as altered GX substitution pattern. Hence, while the precise mechanism is unknown, we conclude that GX is essential to proper cellulose organization in the eudicot SCW and that not only total GX content but also GX DP are critical for proper GX functioning in the SCW.

The complementation mostly reverts the *irx15 irx15-L* mutant phenotype to wild type, but not completely. The SCW composition reverts fully as observed previously by Jensen et al. (2011), but the AFM shows a somewhat irregular cellulose pattern compared to the wild type, while the pattern is more regular than the one found in *irx10*. The nanomechanical properties revert to the wild type except the modulus which further increases in heterogeneity compared to the modulus of any of the three GX mutants. Lastly, the complementation line does not revert the increase in saccharification found for *irx15 irx15-L*. This suggests that *IRX15(-L)* function have not been completely restored by the complementation construct. Brown et al. (2011) found that *irx15* has a stronger mutant phenotype than *irx15-L* and that the increase in cell wall sugar release from *irx15* and *irx15 irx15-L* was the same. Hence, the complementation line appears to be missing a functional *IRX15*, suggesting that *IRX15* and *IRX15-L* are not functionally identical. Alternatively, the promoters of *IRX15* and *IRX15-L* are too distinct for the *IRX15-L* construct to complement the missing *IRX15* expression in the mutant. The latter was found to be the case for both the *IRX9/IRX9-L* and the *IRX10/IRX10-L* gene pairs in primary cell wall xylan biosynthesis using promoter exchange experiments (Mortimer et al., 2015).

It is interesting to note that the AFM of wild type reveals a loosely defined network of fibrillary bundles that merge and split in a hierarchical manner resulting in a continuous size distribution in the range of ~5–50 nm. This architecture likely provides unique physical properties to the cell wall. A similar network architecture is apparent in Zinnia treachery elements (Lacayo et al., 2010) and to some degree thickened parenchyma cell walls from maize (Ding et al., 2014), while being distinct from the fibrillary architecture of primary cell walls of maize (Ding et al., 2014) and onion (Zhang et al., 2016) parenchyma. Spruce SCW displays discrete macrofibrils in tight packing by AFM (Adobes-Vidal et al., 2020), electron microcopy (Donaldson, 2007) and tomography (Reza et al., 2019). Neutron and X-ray scattering results comparing eudicot wood (birch and cherry) to gymnosperm wood (conifer) showed that the microfibril network is less aggregated or spacing within the aggregates is less uniform in dicots (Jarvis, 2018). Our observations by AFM suggest that the latter is the case in that spacing within the aggregates appears less uniform in Arabidopsis.

Changes in cellulose fibril organization are of similar nature (i.e., fibril bundling and orientational dispersion) in the three GX mutants compared to the wild type, while we find the severity of these changes to correlate with increasing GX deficiency. The clear changes in *irx10*, the weakest of the three mutants, highlight how sensitive cellulose network architecture is to the perturbation of GX. Crystalline cellulose content is unaffected in *irx10* and *irx15 irx15-L* under our growth conditions, arguing that cellulose synthesis is not majorly affected. Rather, increased bundling suggests that cellulose self-interactions have been impacted, while increase in orientational dissipation and the appearance of stray bundles suggest that cellulose synthase complex (CSC) movement in the plasma membrane is affected.

It is possible that the sample dehydration involved in the preprocessing of both SEM and AFM could have some effect on the observed cell wall architecture and nanomechanical properties. Given the smoothness and regularity of the cellulose fibrils, the dehydration seems to have a limited effect on the wild type but it is possible that the mutant phenotypes are magnified by dehydration. Several publications suggest that water is bound to the surface of cellulose as well as the hemicelluloses–cellulose interface and that this influences the interactions between the polysaccharides (Hill et al., 2009; Jarvis, 2018; Khodayari et al., 2021). Since the cell wall is already destabilized in the mutants by disorganization, removal of structurally bound water could lead to larger changes in the mutants than in the wild type. This will most likely have the largest effect on local phenomena, such as cellulose bundling, rather than on global phenomena, such as orientational dispersion and stray bundles. Dehydration may also aggravate the differences observed between the wild type and mutants for the adhesion and dissipation in the nanomechanical properties. Increased electrochemical heterogeneity suggests that GX binding to cellulose, including structural water, is significantly different in the mutants. Consequently, some of the structurally bound water may have been maintained between GX and cellulose in the wild type but lost during the desiccation in the mutants. While these characteristics may be magnified by dehydration, we argue that the differences between the wild type and mutants must still be present in the native state in more subtle form, e.g., inhomogeneous GX binding and inhomogeneous cellulose bundling.

Lyczakowski et al. (2019) quantified the width distribution of cellulose bundles in hydrated cryo-preserved SCWs of vessels in Arabidopsis based on fibril bundles perturbing out from wall fractures. We were not able to resolve the finest bundles in our study and therefore unable to assess the bundle width distribution properly. Instead we quantify the distribution of the largest bundles. In the wild type, the bundle width distribution is 10 to 35 nm wide with an average of ~23 nm. Lyczakowski et al. (2019) quantified the width distribution to span ~from 9 to 28 nm with an average of ~17 nm. Our observations are therefore in good agreement with those of Lyczakowski et al. (2019) for the wild type. We also describe and quantify the width of abnormal bundle agglomerates in the mutants, some of these being more than 100 nm wide in *irx9* and *irx15 irx15-L*. Lyczakowski and co-workers found that the fibril width is reduced in both *irx9* and *irx10* compared to the wild type. The difference between the two studies may be because the larger agglomerations visible by imaging the inner side of the SCW do not appear clearly in wall fractures, and these were therefore not characterized by Lyczakowski and co-workers. Also, as the resolution is lower for the mutants than for the wild type in our AFM studies, given the rougher surface of the mutant walls, we cannot assess if the bundle fibrils have changed in width in the mutants compared to the wild type. Our observation of increased bundling is therefore primarily based on the extensive occurrence of larger agglomerates in the mutants and does not exclude that average bundle width of the finer fibrils decrease, as observed by cryo-EM. Our study and that by Lyczakowski et al. (2019) are therefore complementary to each other in characterizing cellulose microfibril width in *irx9* and *irx10*.

The glycome profiling experiment shows increased extractability for GX, pectin, and type II arabinogalactan in *irx9* and *irx15 irx15-L*and thereby indicates a loosened wall structure. The reason behind this may be the increased penetration of the extraction solution in the GX mutants compared to the wild type as the disorganized GX-cellulose network present in the mutants possibly results in a more porous SCW. Alternatively, the phenomena relays on covalent or non-covalent bonds between GX, pectin, and AGPs, which are not formed in the GX mutants leading to a weaker integration of each of these groups of polymers in the wall. An example of the latter for the primary cell wall is APAP1, which is an AGP decorated with arabinogalactan, as well as, xylan and ramnogalacturonan I. The *apap1* mutant that is missing in APAP1 displays increased cell wall extractability (Tan et al., 2013). The work of Biswal et al. (2018) provides further evidence for the presence of xylan-pectin-AGP structures by downregulating homologs of *GAUT4*, a pectin biosynthesis gene, in poplar, rice, and switchgrass and observing cell wall loosening. Therefore, the increased extractability of pectin and arabinogalactanin the GX mutants may suggest that AGPs analogous to APAP1 may also exists for the SCW.

We found a lower RCI in *irx9* and *irx15 irx15-L* by WAXS. This correlates with a 10% reduction in crystalline cellulose in *irx9* determined by chemical analysis, while crystalline cellulose content in *irx15 irx15-L* is unchanged. Removal of matrix polysaccharides by mild chemical extraction leads to a concurrent increase in the crystalline cellulose content but a reduction in RCI, which is surprising. One possible explanation is that GX bound to cellulose may extend the crystallite size by binding in the linear two-fold helical screw conformation. If so, the removal of GX from the biomass during the carbonate extraction could lead to a reduction of the xylan–cellulose crystallite size leading to a reduced RCI. Alternatively, it is the changes in cellulose organization in *irx15 irx15-L*, which may also be the case in the wild type material after carbonate extraction that somehow leads to reduced RCI. As a result, not only cellulose content and crystallinity, but also the binding of hemicellulose and organizational changes in the cellulose fibril network, may lead to changes in RCI.

Several publications document increased saccharification of plant material with impaired xylan biosynthesis. For instance, the *irx9* and *irx15 irx15-L* mutations in Arabidopsis lead to 39% (Petersen et al., 2012) and 46% (Brown et al., 2011) increase in saccharification, respectively, whereas RNAi of homologs of *IRX7, IRX8, IRX9*, and *IRX10* in rice or poplar resulted in increased saccharification by 25–50% in most cases (Lee et al., 2009; Chen et al., 2013; Biswal et al., 2015; Ratke et al., 2018). As pointed out in many of these studies, increased saccharification of xylan deficient plants indicates an altered association of xylan with cellulose in the cell wall material. In our study, we provide a direct comparison between *irx9, irx10*, and *irx15 irx15-L* in a saccharification assay. The increases in sugar release for each of the mutants are comparable to previous observations and correlate with the degree of mutant severity. Because we chose no pretreatment, it is possible to directly link increases in Glc and Xyl release with the architectural changes observed by SEM and AFM at the nano- and microscale. As the increases in release are approximately equal for Glc and Xyl for each of the mutants, it underlines the structural interdependence that exists in the SCW between GX and cellulose.

### GX as a Modulator of Fibril Coalescence and Bundle Formation

Our studies show that uniform cellulose fibril bundling and alignment is disrupted in the GX mutants. We also observe larger fibrillary agglomerations and larger crevasses in the weaving pattern. These observations may suggest that fibril coalescence is increased or uncontrolled in the mutants. If so, it suggests that GX functions by modulating microfibril coalescence and acts *in planta* to regulate cellulose bundling and network architecture during wall assembly. This general idea concerning hemicellulose function was first formulated several decades ago as part of a series of seminal studies with bacterial cellulose produced in the presence of various hemicelluloses (Haigler et al., 1982; Atalla et al., 1993; Tokoh et al., 1998, 2002). Other *in vitro* studies with holocellulose (delignified cellulose with native hemicellulose still bound) have since shown that hemicellulose bound to cellulose in native form does indeed effectively prevent cellulose coalescence (Iwamoto et al., 2008; Arola et al., 2013; Yang et al., 2020). Results similar to ours, but for the primary cell wall, were found investigating the *xxt1xxt2* Arabidopsis mutant (Anderson et al., 2010; Xiao et al., 2016). This mutant is entirely deficient in xyloglucan and displays increased cellulose bundling and altered fibril alignment in the primary cell wall leading the authors to suggest that xyloglucan may function to reduce spontaneous interactions of cellulose microfibrils (Xiao et al., 2016), i.e., fibril coalescence. In-depth studies characterizing cellulose organization in hemicellulose mutants, such as the *xxt1xxt2* studies as well as our own present study on GX mutants, are still few and serve as an important complement to the large body of *in vitro* experiments on cellulose–hemicellulose interactions from the past decades.

In conclusion, a growing body of experimental evidence suggests that some hemicelluloses under some conditions may play a crucial role in modulating cellulose fibril coalescence *in vitro*, as well as, *in planta*. Our study suggests that microfibril coalescence is not only important in primary cell walls, as shown previously, but also in SCWs and that GX performs a key role in this in Arabidopsis.

## Data Availability Statement

The original contributions presented in the study are included in the article/Supplementary Material, further inquiries can be directed to the corresponding author/s.

## Author Contributions

DH and JJ conceived the project. JC, PH, SP, and HP conducted the experiments under the supervision of S-YD, DH, and JJ. JC, S-YD, DH, and JJ analyzed the data. JC and JJ wrote the paper. All authors contributed to the article and approved the submitted version.

## Funding

JC was supported in part by a grant from NSF (NSF CBET 1336622). JJ was supported by EU grant HRZ 2020 Marie Sklodowska-Curie Actions (MSCA IF 841703). S-YD was supported by the US Department of Energy, Office of Science, Office of Biological and Environmental Research (DE-SC0019072), and S-YD and DH were supported by the Great Lakes Bioenergy Research Center (DE-SC0018409).

## Conflict of Interest

The authors declare that the research was conducted in the absence of any commercial or financial relationships that could be construed as a potential conflict of interest.

## Publisher's Note

All claims expressed in this article are solely those of the authors and do not necessarily represent those of their affiliated organizations, or those of the publisher, the editors and the reviewers. Any product that may be evaluated in this article, or claim that may be made by its manufacturer, is not guaranteed or endorsed by the publisher.
