# Supplementary material for: Xylan Is Critical for Proper Bundling and Alignment of Cellulose Microfibrils in Plant Secondary Cell Walls
Source: Front Plant Sci. 2021 Sep 23;12:737690. doi: 10.3389/fpls.2021.737690 (PMC8495263; doi:10.3389/fpls.2021.737690)
Supplement: Supplementary file 1 [file Presentation_1.PPTX]

## Slide 1
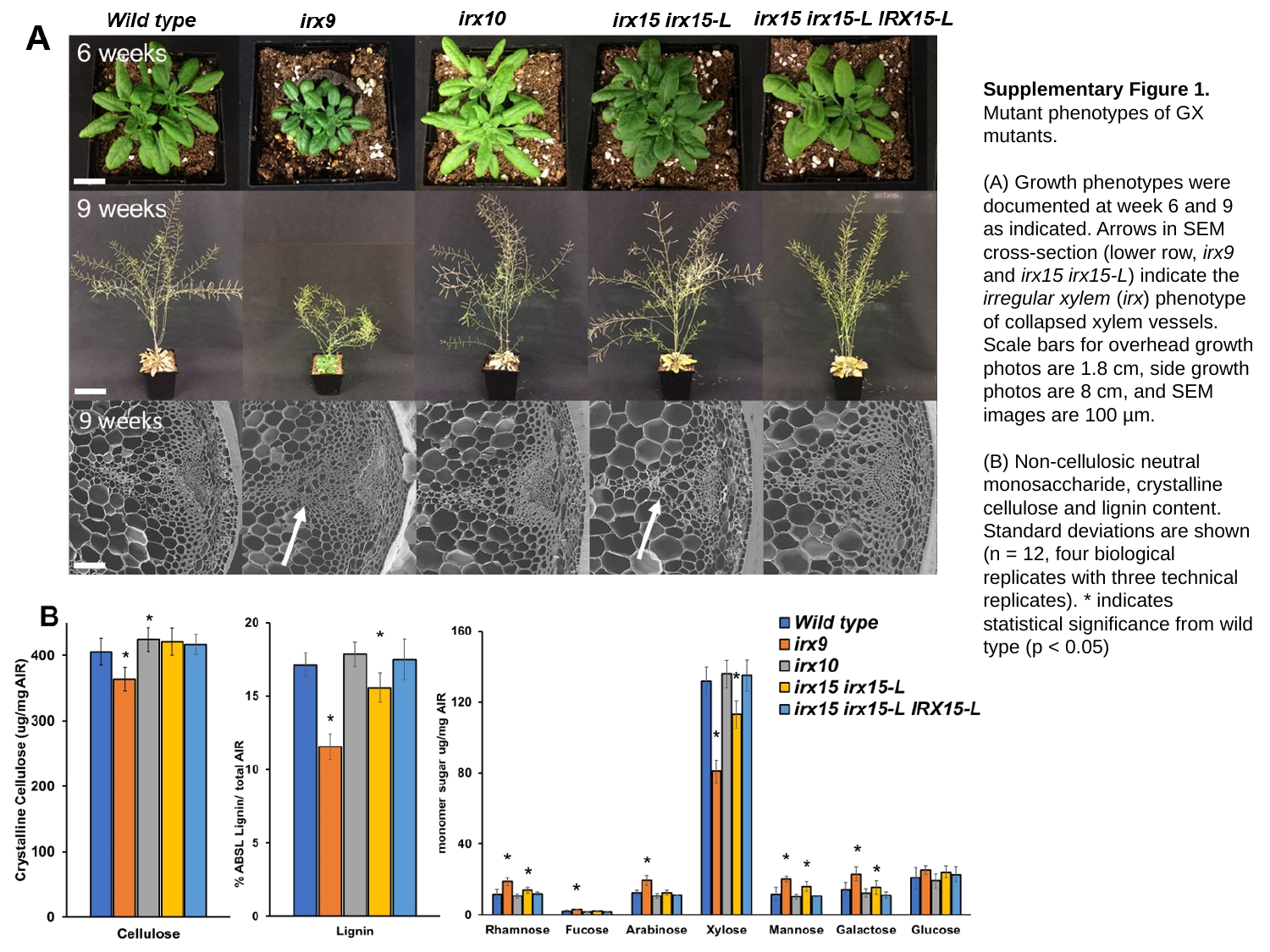

Supplementary Figure 1. Mutant phenotypes of GX mutants.
(A) Growth phenotypes were documented at week 6 and 9 as indicated. Arrows in SEM cross-section (lower row, irx9 and irx15 irx15-L) indicate the irregular xylem (irx) phenotype of collapsed xylem vessels. Scale bars for overhead growth photos are 1.8 cm, side growth photos are 8 cm, and SEM images are 100 µm.
(B) Non-cellulosic neutral monosaccharide, crystalline cellulose and lignin content. Standard deviations are shown (n = 12, four biological replicates with three technical replicates). * indicates statistical significance from wild type (p < 0.05)

## Slide 2
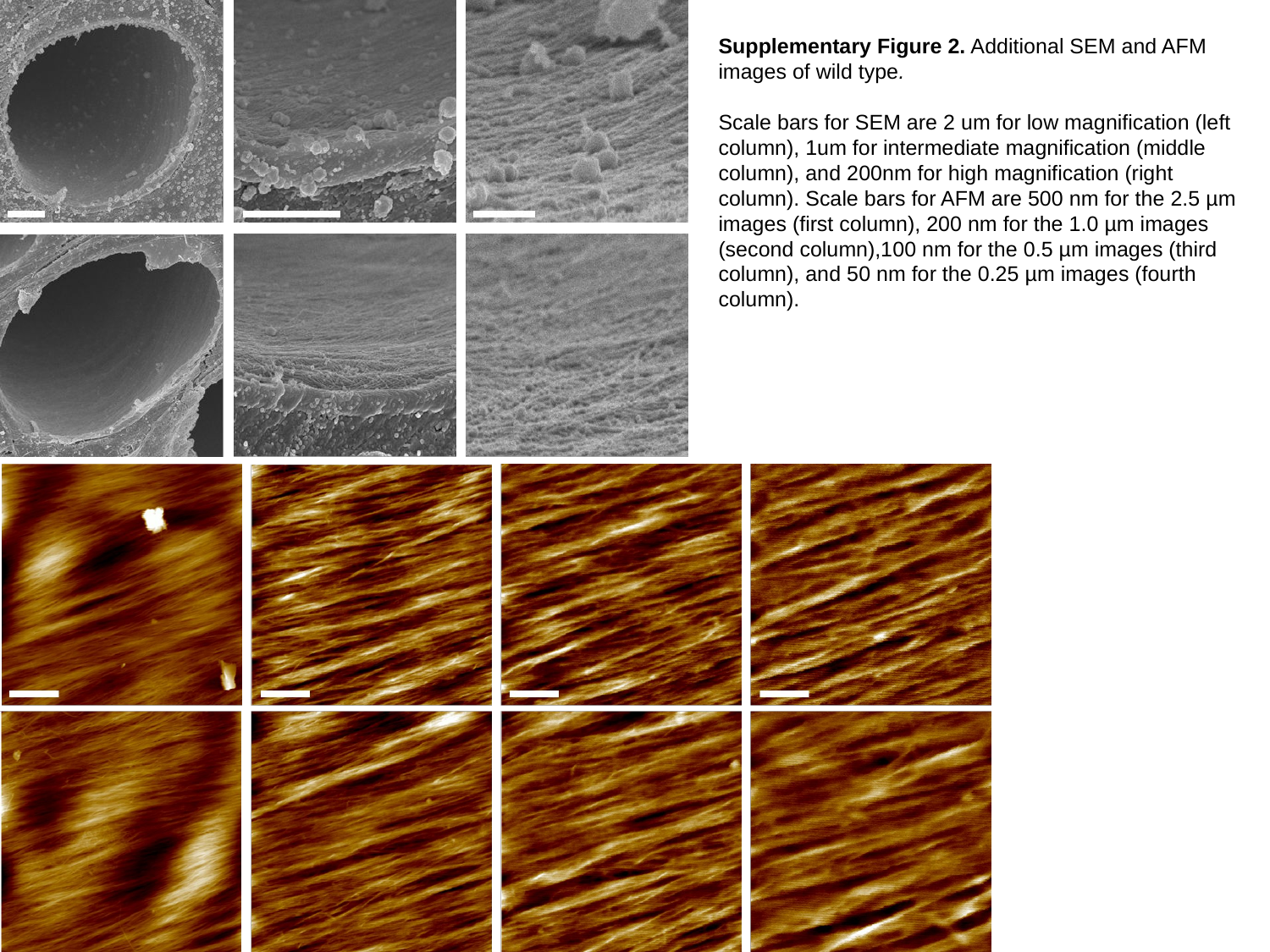

Supplementary Figure 2. Additional SEM and AFM images of wild type.
Scale bars for SEM are 2 um for low magnification (left column), 1um for intermediate magnification (middle column), and 200nm for high magnification (right column). Scale bars for AFM are 500 nm for the 2.5 µm images (first column), 200 nm for the 1.0 µm images (second column),100 nm for the 0.5 µm images (third column), and 50 nm for the 0.25 µm images (fourth column).

## Slide 3
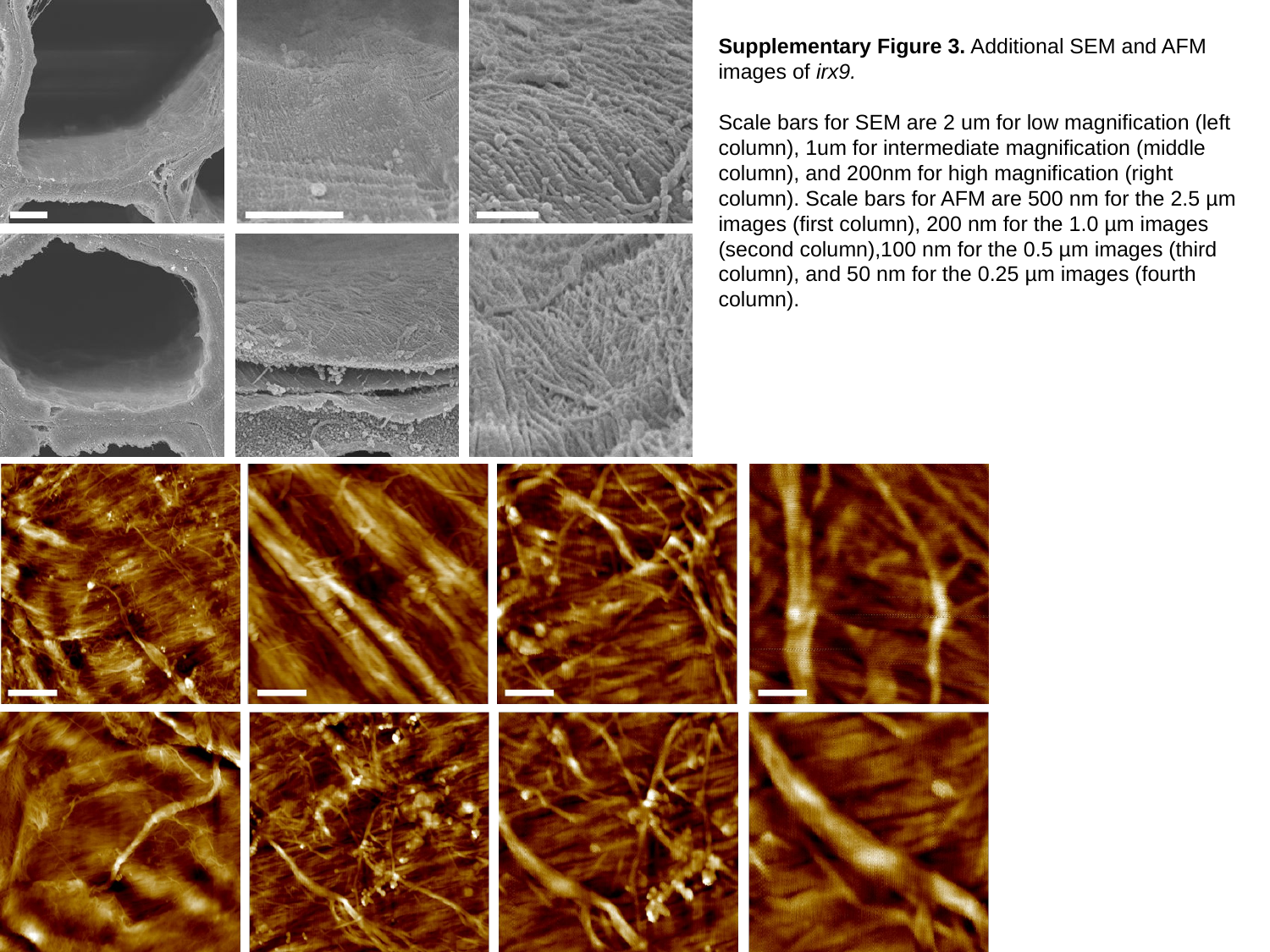

Supplementary Figure 3. Additional SEM and AFM images of irx9.
Scale bars for SEM are 2 um for low magnification (left column), 1um for intermediate magnification (middle column), and 200nm for high magnification (right column). Scale bars for AFM are 500 nm for the 2.5 µm images (first column), 200 nm for the 1.0 µm images (second column),100 nm for the 0.5 µm images (third column), and 50 nm for the 0.25 µm images (fourth column).

## Slide 4
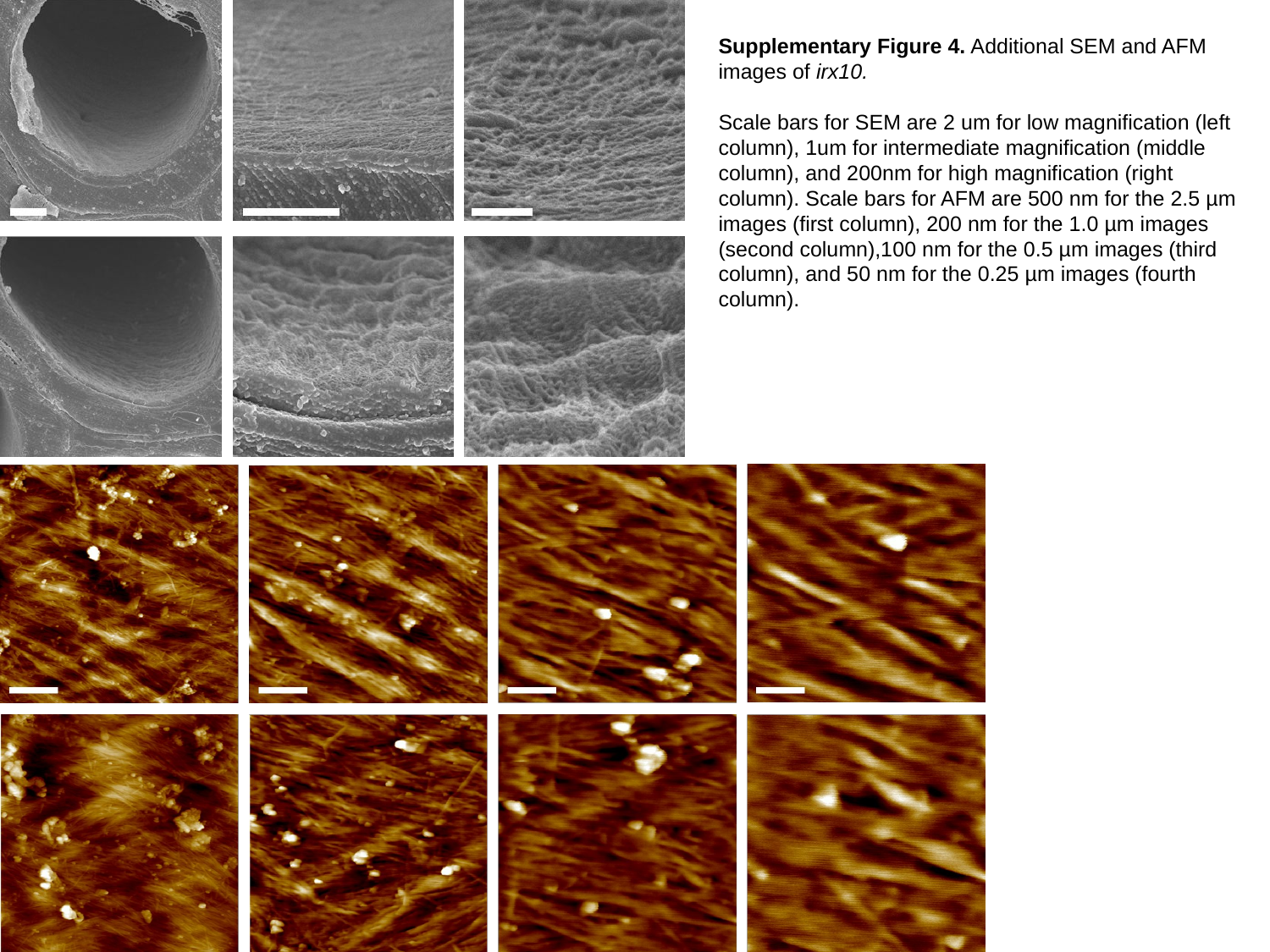

Supplementary Figure 4. Additional SEM and AFM images of irx10.
Scale bars for SEM are 2 um for low magnification (left column), 1um for intermediate magnification (middle column), and 200nm for high magnification (right column). Scale bars for AFM are 500 nm for the 2.5 µm images (first column), 200 nm for the 1.0 µm images (second column),100 nm for the 0.5 µm images (third column), and 50 nm for the 0.25 µm images (fourth column).

## Slide 5
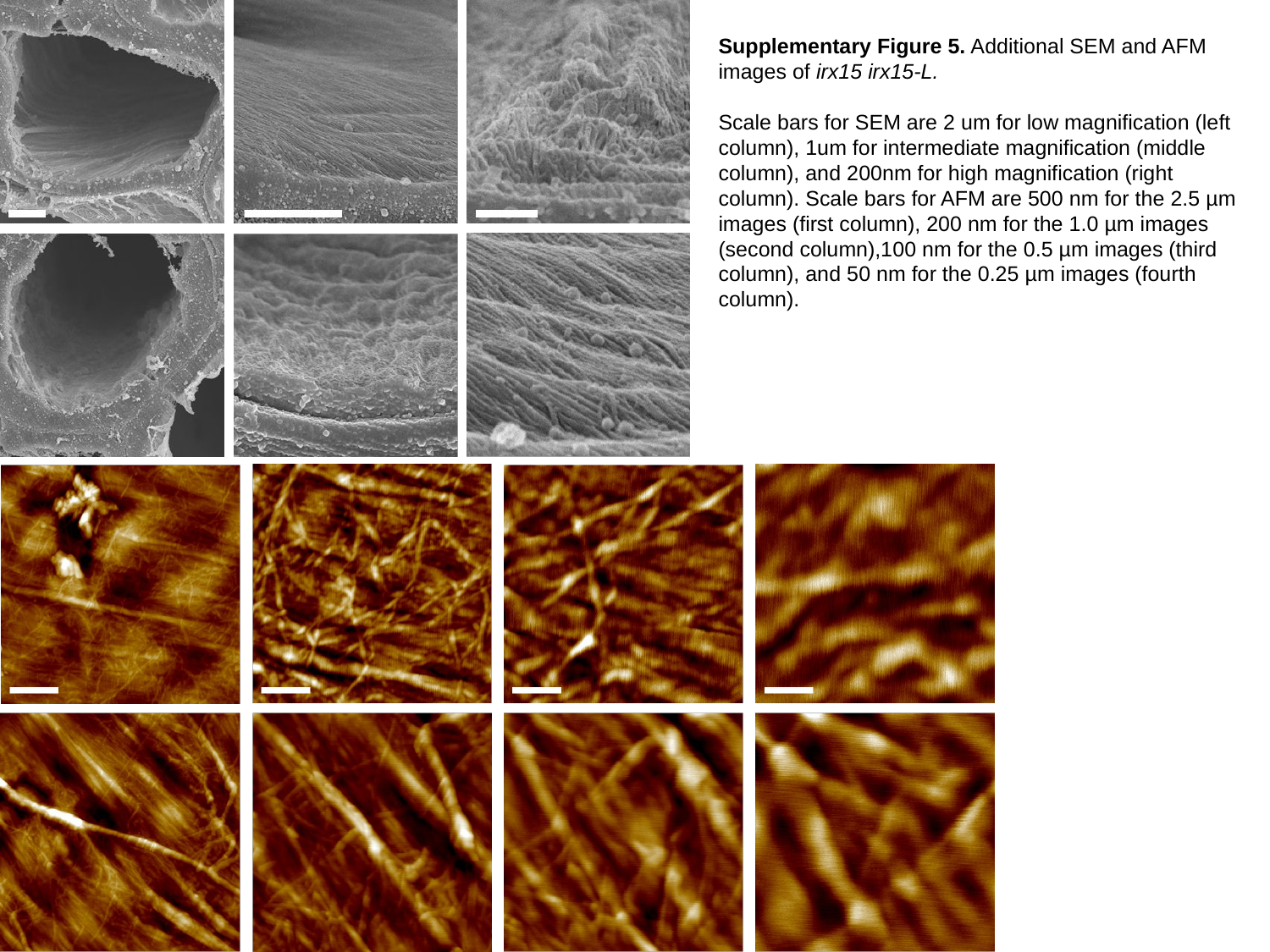

Supplementary Figure 5. Additional SEM and AFM images of irx15 irx15-L.
Scale bars for SEM are 2 um for low magnification (left column), 1um for intermediate magnification (middle column), and 200nm for high magnification (right column). Scale bars for AFM are 500 nm for the 2.5 µm images (first column), 200 nm for the 1.0 µm images (second column),100 nm for the 0.5 µm images (third column), and 50 nm for the 0.25 µm images (fourth column).

## Slide 6
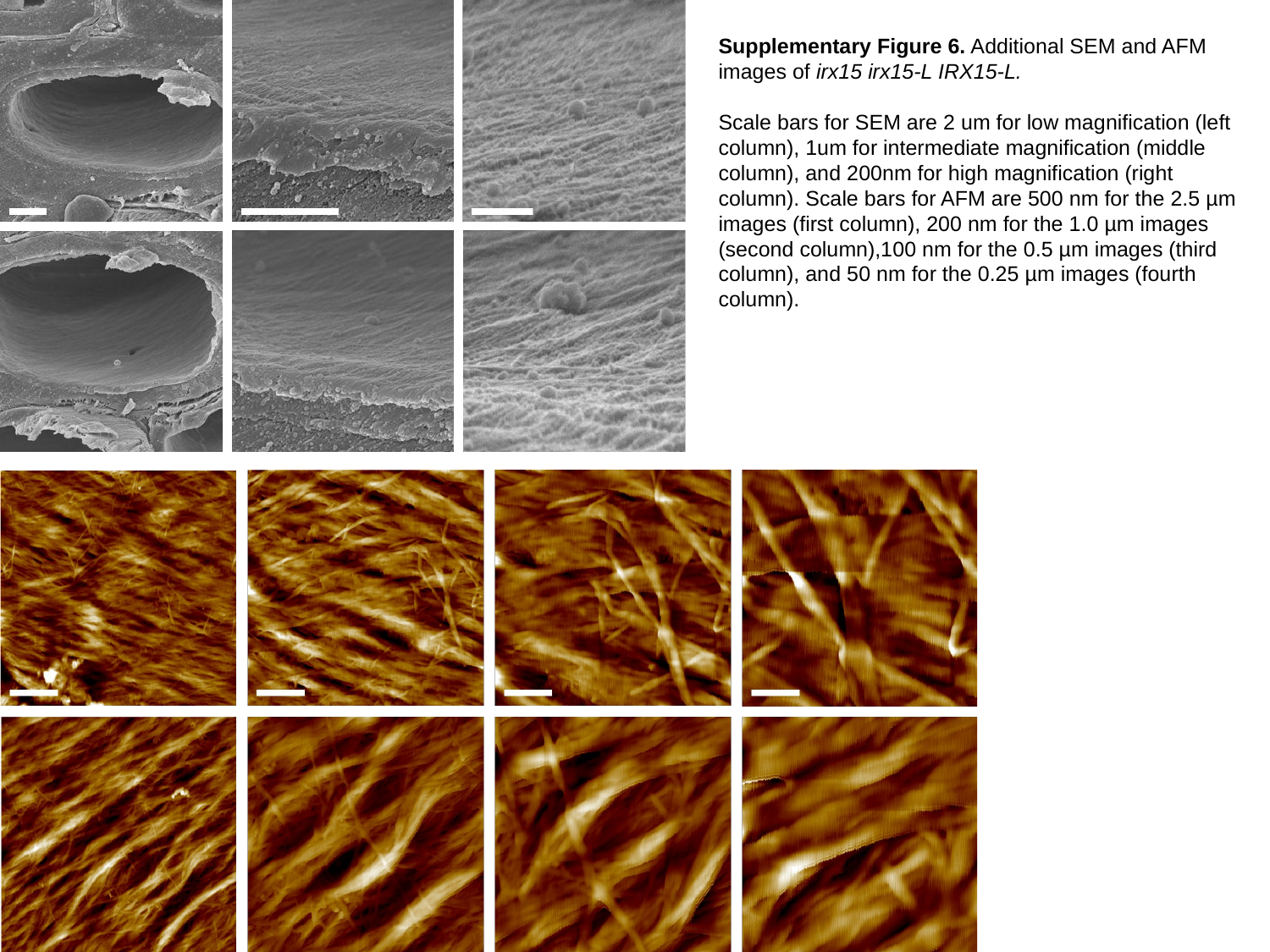

Supplementary Figure 6. Additional SEM and AFM images of irx15 irx15-L IRX15-L.
Scale bars for SEM are 2 um for low magnification (left column), 1um for intermediate magnification (middle column), and 200nm for high magnification (right column). Scale bars for AFM are 500 nm for the 2.5 µm images (first column), 200 nm for the 1.0 µm images (second column),100 nm for the 0.5 µm images (third column), and 50 nm for the 0.25 µm images (fourth column).

## Slide 7
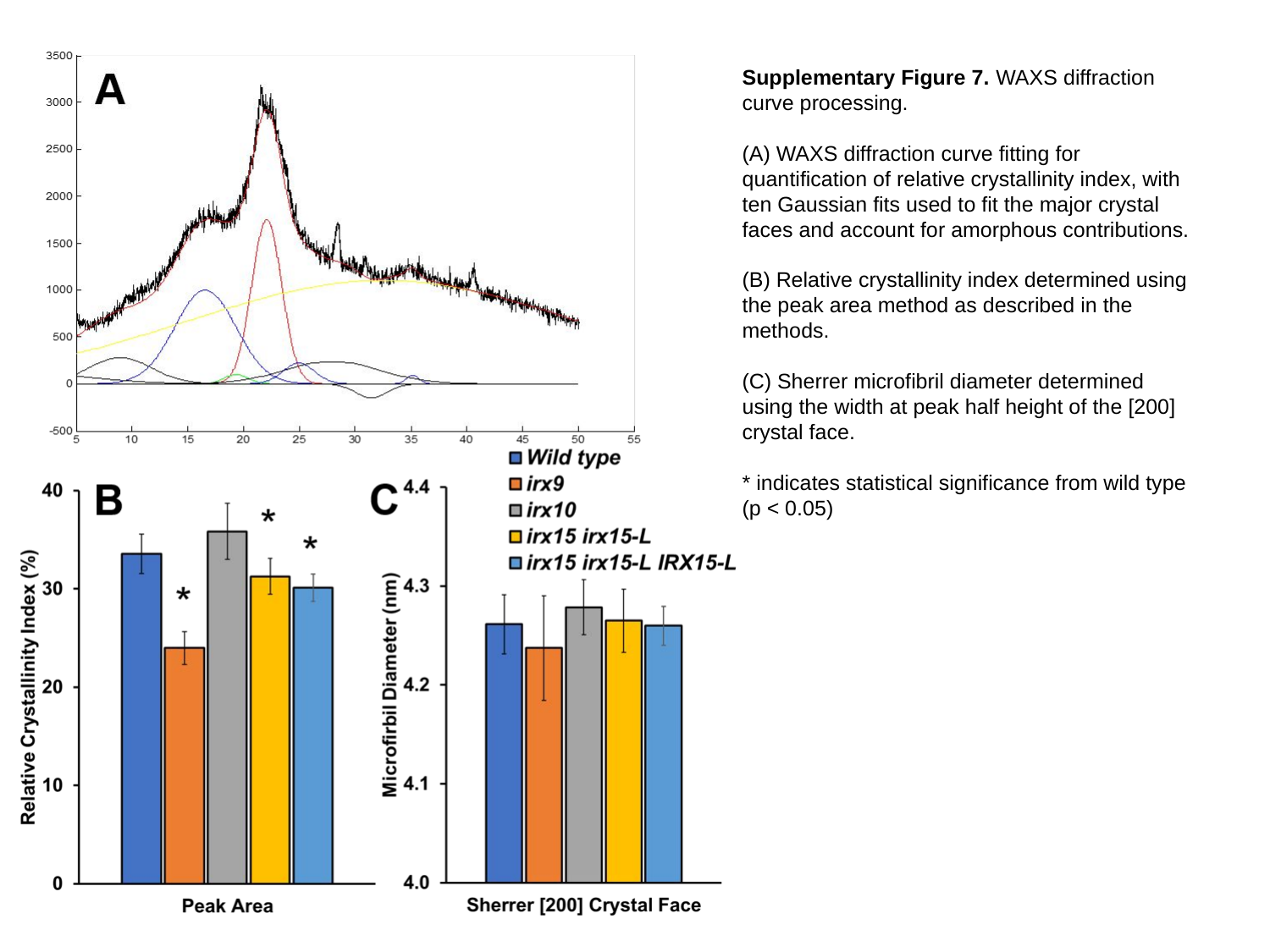

Supplementary Figure 7. WAXS diffraction curve processing.
(A) WAXS diffraction curve fitting for quantification of relative crystallinity index, with ten Gaussian fits used to fit the major crystal faces and account for amorphous contributions.
(B) Relative crystallinity index determined using the peak area method as described in the methods.
(C) Sherrer microfibril diameter determined using the width at peak half height of the [200] crystal face.
* indicates statistical significance from wild type (p < 0.05)
