# Supplementary material for: Xylan Is Critical for Proper Bundling and Alignment of Cellulose Microfibrils in Plant Secondary Cell Walls
Source: Front Plant Sci. 2021 Sep 23;12:737690. doi: 10.3389/fpls.2021.737690 (PMC8495263; doi:10.3389/fpls.2021.737690)
Supplement: Supplementary file 2 [file Presentation_2.PPTX]

## Slide 1
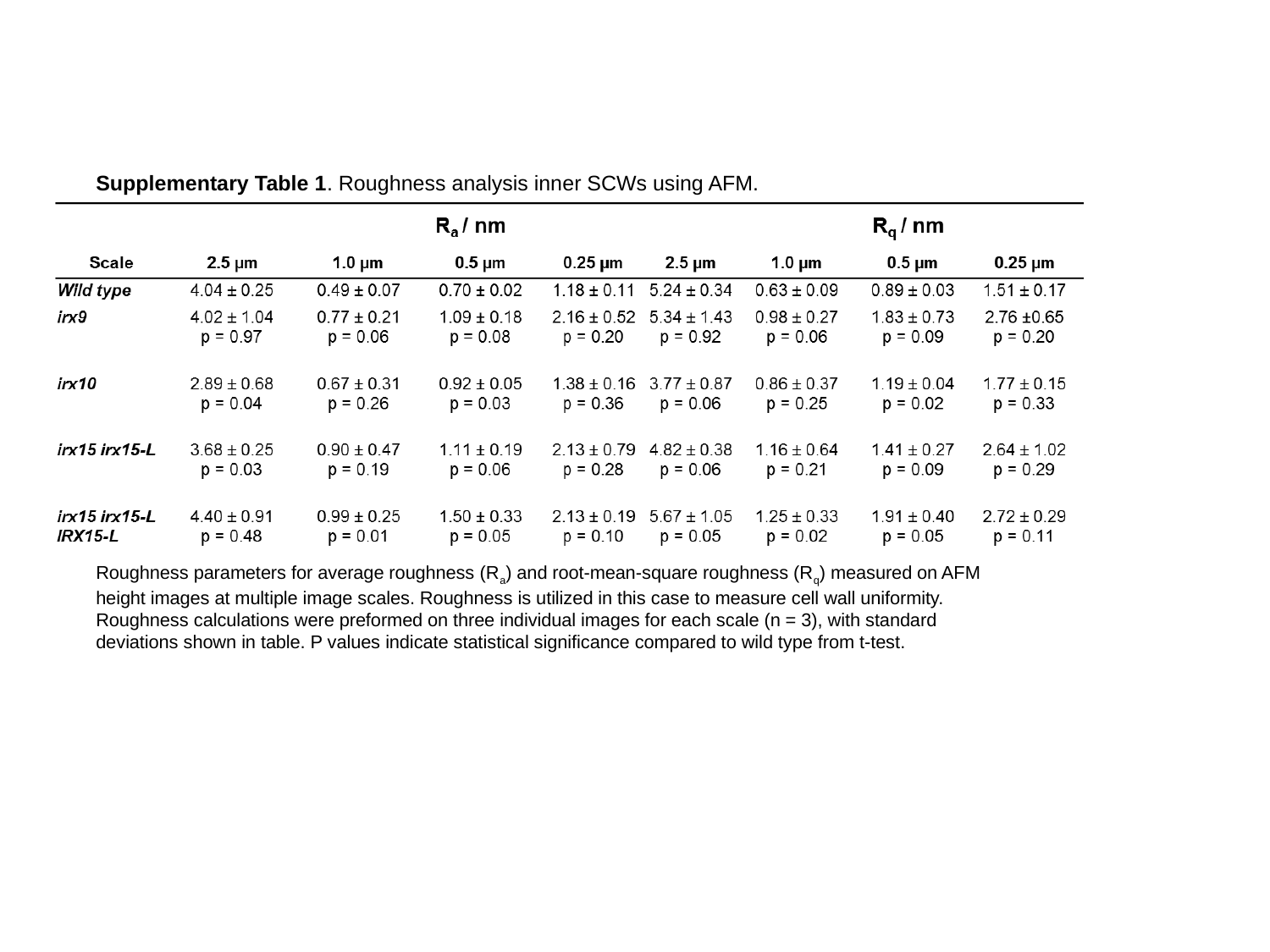

Supplementary Table 1. Roughness analysis inner SCWs using AFM.
Roughness parameters for average roughness (Ra) and root-mean-square roughness (Rq) measured on AFM height images at multiple image scales. Roughness is utilized in this case to measure cell wall uniformity. Roughness calculations were preformed on three individual images for each scale (n = 3), with standard deviations shown in table. P values indicate statistical significance compared to wild type from t-test.
